# VCAM-1–targeting peptide assemblies protect vascular endothelium and prolong cardiac xenograft survival

**DOI:** 10.1126/sciadv.aec5707

**Published:** 2026-07-08

**Authors:** Yi-Jing Li, Hang Zhang, Zhe Sun, Kai Xing, Xiumeng Hua, Peiyuan Li, Xiao Chen, Han Mo, Jiangping Song

**Affiliations:** ^1^ Shenzhen State Key Laboratory of Cardiovascular Disease, Fuwai Hospital, Chinese Academy of Medical Sciences, Shenzhen 518057, China.; ^2^ State Key Laboratory of Cardiovascular Disease, Fuwai Hospital, National Center for Cardiovascular Diseases, Chinese Academy of Medical Sciences and Peking Union Medical College, Beijing 100037, China.; ^3^ Clinical Research Institute, Chinese Academy of Medical Sciences, Faculty of Clinical Medicine, Peking Union Medical College, Beijing 100730, China.; ^4^ Sanya Institute of China Agricultural University, Sanya, 572024, China.; ^5^ Beijing Key Laboratory of Xenotransplantation, Fuwai Hospital, National Centre for Cardiovascular Disease, Chinese Academy of Medical Sciences and Peking Union Medical College, Beijing 100037, China.

## Abstract

Cardiac xenotransplantation represents a promising strategy to address the shortage of donor hearts, yet endothelial cell dysfunction remains a major obstacle to long-term graft survival. Using integrated single-cell RNA sequencing in a porcine-to-primate xenotransplantation model, we identified a VCAM-1^+^ endothelial subpopulation as the primary endothelial subtype susceptible to acute rejection, characterized by its selective depletion after transplantation. To protect this population, we develop VCAM-1–targeted nanoparticles (VTNs) integrating three functional modules: (i) a VCAM-1–binding peptide identified, (ii) a self-assembling peptide module by one-bead one-compound screening for precise targeting that forms protective nanofibrous coatings, and (iii) localized delivery of mycophenolate mofetil (MMF) for immune modulation. VTN reduces immune cell adhesion by 73% (*P* < 0.001) and extends xenograft survival nearly fourfold, from 6.7 to 27.0 days. These findings establish VCAM-1^+^ endothelial cells as a therapeutic target and highlight VCAM-1–targeted nanomedicine as a promising approach to improve xenograft outcomes.

## Teaser

VTN system protects cardiac xenografts by creating a defensive barrier on vessels, addressing the critical donor heart shortage.

## INTRODUCTION

Cardiac xenotransplantation (CXTx) has emerged as a critical solution to address the dire global shortage of donor hearts, yet immune-mediated endothelial injury remains the primary barrier to long-term xenograft survival (*1*, *2*). Vascular endothelial cells (ECs), occupying the crucial interface between circulating immunity and the graft, orchestrate vascular homeostasis, thromboresistance, and innate immune regulation (*3*, *4*). CXTx faces amplified challenges beyond allograft settings: Species-specific molecular incompatibilities (notably, α-Gal and Neu5Gc xenoantigens) trigger explosive innate immune activation, which exacerbates classical ischemia-reperfusion injury and adaptive alloimmunity (*5*, *6*). While multigene editing and combinatorial immunosuppressive regimens have been developed to mitigate these challenges (*7*, *8*), complete prevention of EC injury remains elusive. Critically, posttransplant degradation of the endothelial glycocalyx exposes adhesion molecules such as vascular cell adhesion molecule–1 (VCAM-1) (*2*, *9*), initiating proinflammatory cascades that recruit innate immune cells and accelerate rejection. Conventional immunosuppressants like mycophenolate mofetil (MMF), although effective against T cell responses, fail to address the intrinsic vulnerability of xenogeneic endothelium and may further compromise EC repair mechanisms (*10*–*12*). This therapeutic gap underscores the urgent need for targeted strategies capable of concurrently preserving endothelial integrity and modulating local immune responses (*12*–*27*).

**Fig. 1.**
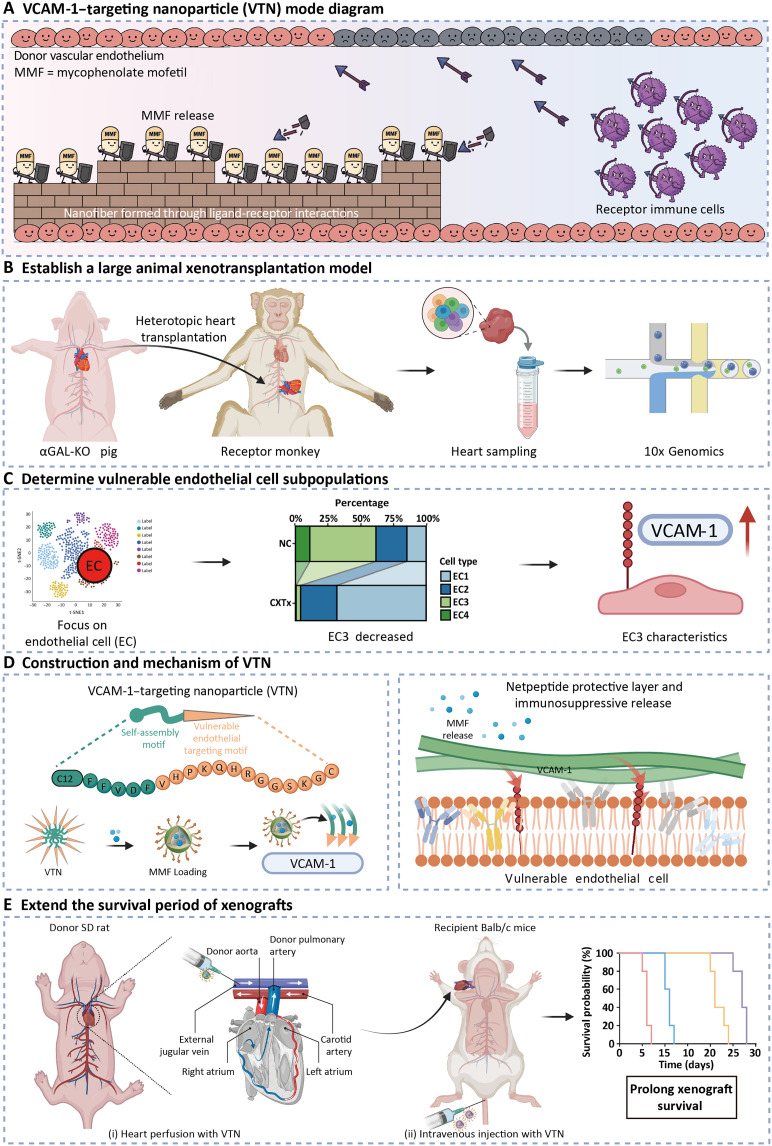
Schematic of VTN nanomaterial system for VCAM-1–targeted endothelial protection in CXTx. (**A**) Self-assembled peptide nanofibers form a physical nanofiber wall on endothelial surfaces, whereas encapsulated MMF provides localized immunosuppression to repel infiltrating immune cells. (**B**) Experimental framework and VTN design: Xenotransplantation model: α-1,3-galactosyltransferase gene knockout (GTKO) porcine hearts were transplanted into macaque recipients. (**C**) Myocardial tissues were analyzed via 10x Genomics scRNA-seq to identify vulnerable VCAM-1^+^ ECs. t-SNE, t-Distributed Stochastic Neighbor Embedding. (**D**) VTN molecular structure: VTN consists of a self-assembly motif (C12-FFVDF) and a VCAM-1–targeting sequence (VHPKQHRGGGSKGC), forming nanoparticles that bind specifically to VCAM-1 on ECs and release MMF to suppress local immunity. (**E**) Therapeutic applications: (i) Donor hearts were perfused with VTN via the aorta and pulmonary artery before transplantation; (ii) in the rat-to-mouse cardiac xenograft model, VTN was administered via the jugular vein to prolong graft survival. Created in BioRender. Y. Li, (2026) https://BioRender.com/o2invd9.

To overcome this dual challenge, preserving endothelial integrity while controlling local immunity, we conceived a defense strategy hierarchical architecture. Leveraging single-cell RNA sequencing (scRNA-seq) in a porcine-to-primate CXTx model, we identified the selective depletion of VCAM-1^+^ EC subpopulation as a hallmark of acute rejection, establishing their pretransplant VCAM-1 expression as an ideal anchoring site for prophylactic intervention. We engineered VCAM-1–targeted nanoparticles (VTNs) with tripartite functionality: (i) VCAM-1–targeting peptide for precise EC delivery; (ii) self-assembling peptides that form nanofibrous barriers shielding endothelial adhesion molecules; and (iii) MMF loading, local immunosuppression (Fig. 1).

We validated this system in rat-to-mouse cardiac xenografts, and this bioinspired VTN system significantly reduces immune cell adhesion, prolongs xenograft survival, and potentially minimizes systemic immunosuppressive toxicity. These findings establish a paradigm for endothelial protection in CXTx while extending graft survival. Beyond validating VCAM-1 as a therapeutic target, this work pioneers the application of architectural biomimicry in transplant medicine, offering a combinatorial strategy to overcome limitations of current immunosuppressive approaches.

## RESULTS

### VCAM-1^+^ ECs represent the predominant injured EC subtype in CXTx

**Fig. 2.**
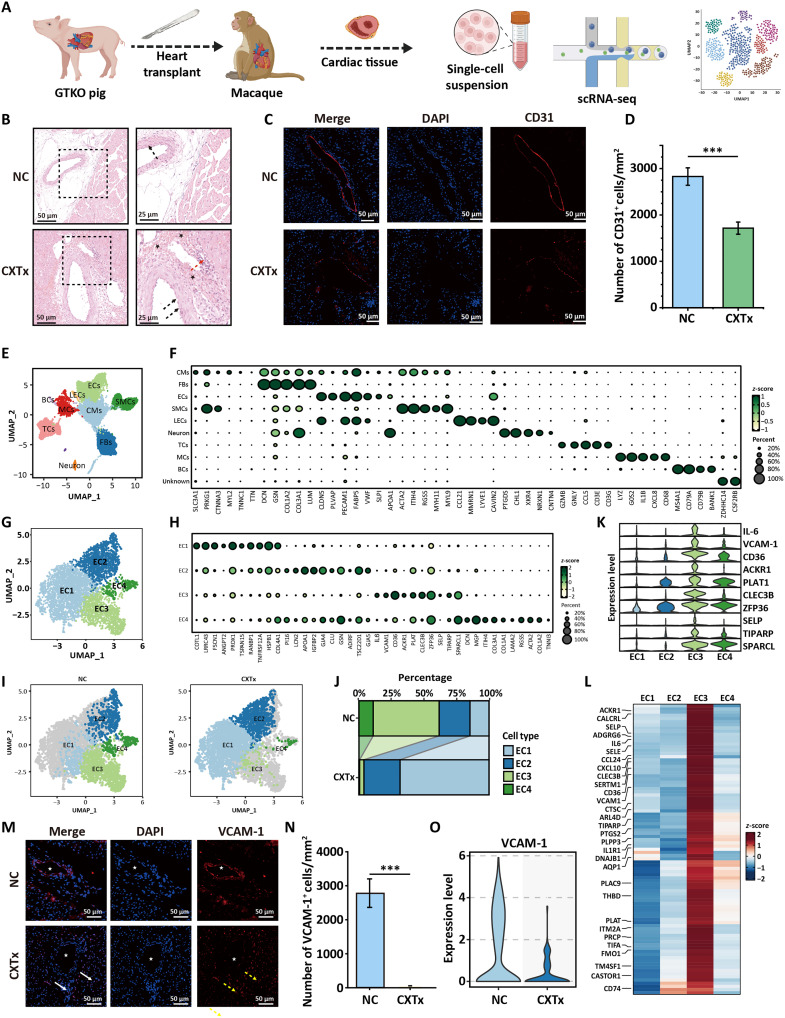
Single-cell transcriptomics and histopathology identify VCAM-1^+^ EC3 as the vulnerable endothelial subtype in CXTx. (**A**) Experimental workflow. (**B**) Hematoxylin and eosin (H&E) staining showing EC injury and inflammation in CXTx. (**C** and **D**) CD31 immunofluorescence and quantification (***P < 0.001). (**E** and **F**) UMAP and marker gene bubble plot of major cell types. CMs, cardiomyocytes; FBs, fibroblasts; SMCs, smooth muscle cells; LECs, lymphatic ECs; TCs, T cells; MCs, myeloid cells; BCs, B cells. (**G** to **I**) UMAP of EC subclusters in NC and CXTx. (**J**) EC subtype proportions. (**K** and **L**) Marker gene expression (bubble plot and heatmap) for EC subsets. (**M** to **O**) VCAM-1 immunofluorescence, quantification (***P < 0.001), and violin plot showing reduced expression in CXTx. (A) Created in BioRender. Y. Li, (2026) https://BioRender.com/o2invd9.

To investigate endothelial injury mechanisms in CXTx, we established a heterotopic CXTx model by engrafting α-1,3-galactosyltransferase gene knockout (GTKO) porcine hearts into the abdominal cavity of macaque monkeys. Hearts were harvested at graft failure for pathological examination and scRNA-seq, with nontransplanted GTKO pig hearts serving as normal controls (NCs) (Fig. 2A). Histopathological examination revealed severe vascular EC swelling and detachment in CXTx grafts compared with NC, accompanied by extensive perivascular immune cell infiltration and myocardial damage (Fig. 2B). Immunofluorescence analysis of the endothelial marker CD31 demonstrated continuous, high-intensity signal in NC vessels, confirming intact endothelium. In contrast, CXTx hearts exhibited discontinuous CD31 expression with significantly reduced fluorescence intensity (*P* < 0.001), indicating profound structural disruption of the endothelial layer (Fig. 2C). The CD31^+^ cell counts in the NC and CXTx groups are shown in Fig. 2D.

To characterize injured EC subpopulations, we performed scRNA-seq on myocardial tissues from the NC and CXTx groups, which identified nine major cell clusters: cardiomyocytes, fibroblasts, ECs, smooth muscle cells, lymphatic ECs, neurons, T cells, myeloid cells, and B cells (Fig. 2, E and F). Subclustering of ECs resolved four distinct subtypes (EC1 to EC4) (Fig. 2, G to I). In NC hearts, EC3 constituted the predominant EC subtype, accounting for 50% of all ECs, followed by EC2, whereas EC1 and EC4 were minor populations. Post-CXTx, the proportion of EC3 significantly decreased, suggesting its vulnerability to xenotransplantation-associated injury (Fig. 2J). Differential gene expression analysis demonstrated that EC3 specifically expressed adhesion molecules (VCAM-1) and inflammation-related regulatory genes [interleukin-6 (IL-6) and Atypical Chemokine Receptor 1 (ACKR1)] (Fig. 2, K and L). Both immunofluorescence staining and scRNA-seq data confirmed robust VCAM-1 expression in ECs of NC hearts. Notably, the expression of VCAM-1 in EC was markedly down-regulated post-CXTx (Fig. 2, M to O). Collectively, these findings identify EC3, a population characterized by high baseline expression of VCAM-1, as the EC subtype most vulnerable to CXTx injury. To validate the translational potential of VCAM-1 targeting, we demonstrated >70% sequence homology in VCAM-1 across pig, human, and rodent species, validating our subsequent use of human umbilical vein ECs (HUVEC) and rat-to-mouse transplantation models to evaluate VCAM-1–targeted therapeutics, as conserved target engagement ensures translational relevance to porcine-to-primate xenotransplantation (fig. S2).

### Self-assembly and fibrillar transformation of peptide nanoparticle

**Fig. 3.**
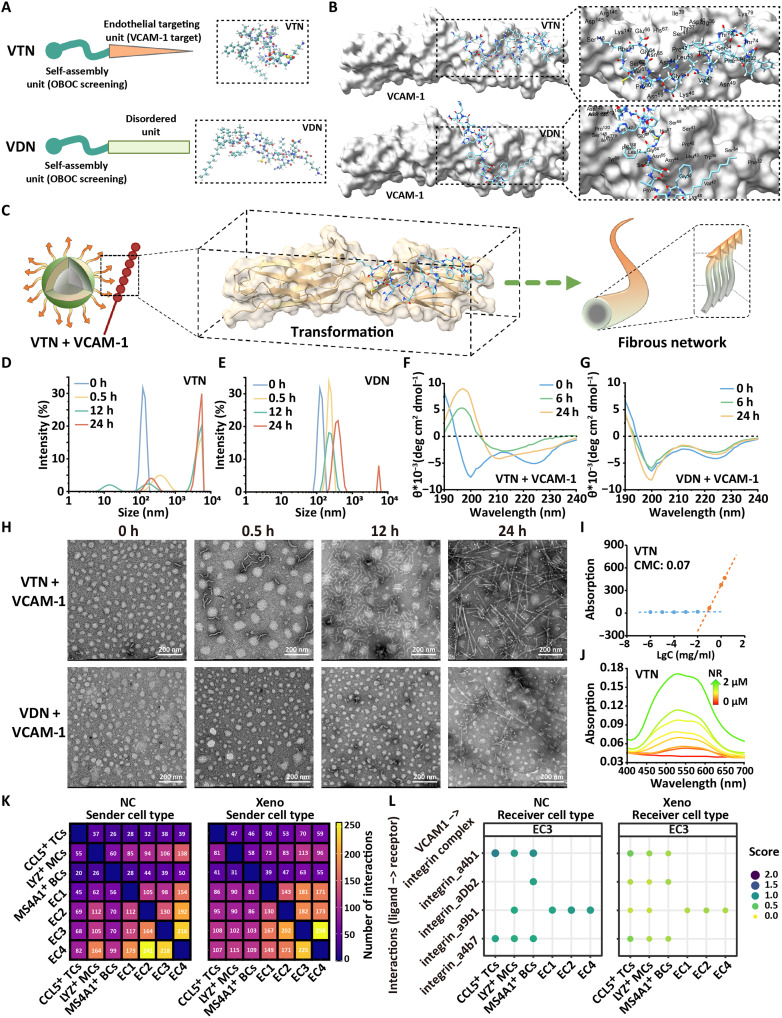
VCAM-1–triggered structural transformation of VTN. (**A**) Molecular structures of VTN and VDN. OBOC, one-bead one-compound. (**B**) Docking of VTN with VCAM-1. (**C**) Schematic of nanoparticle-to-nanofiber transformation. (**D** and **E**) Time-dependent dynamic light scattering (DLS) size distributions of VTN and VDN ± VCAM-1. (**F** and **G**) CD spectra showing β sheet formation in VTN. (**H**) TEM images of VTN and VDN transformation over time (scale bars, 200 nm). (**I**) CMC determination. (**J**) Nile Red (NR) encapsulation by VTN. (**K**) Cell-cell interaction heatmaps in normal control (NC) versus xenotransplanted (Xeno) hearts. (**L**) Integrin–VCAM-1 interaction dot plots demonstrating reduced adhesion signaling in Xeno. h, hours.

To characterize the VCAM-1–triggered structural transformation of our VTN, we first established the molecular architecture of its core components: (i) the VCAM-1–targeting peptide (VHPKQHRGGSKGC) and (ii) the active nanoparticle named VTN (C12-FFVDF-VHPKQHRGGSKGC) integrating lauric acid–derived hydrophobicity with diphenylalanine self-assembly motifs (Fig. 3A and figs. S3 to S6). Nontargeting nanoparticle named VDN (C12-FFVDF-GCHPGSVKRGQHK) lacking VCAM-1 affinity served as control (figs. S7 and S8).

Molecular docking (AutoDock Vina) confirmed high-affinity binding between VTN (the VCAM-1–targeting nanoparticle) and VCAM-1, with a binding energy (Δ*G*) of −8.3 ± 0.4 kcal/mol (Fig. 3B). Figure 3C illustrates the VCAM-1–induced transformation of VTN from nanoparticles into nanofibers with an average diameter of 15 nm. The VCAM-1–mediated transformation of VTN was investigated using dynamic light scattering (DLS) and transmission electron microscopy (TEM). At 37°C, we observed the formation of nanofibers with an average diameter of 10 nm within 0.5 hours in the presence of VCAM-1, whereas control nanoparticles lacking the targeting unit maintained their particulate structure under identical conditions. Following 0.5 hours of incubation with VCAM-1 at 37°C, the average hydrodynamic diameter of VTN shifted from 50.9 nm to a polydisperse distribution (220.0 nm and 1 μm), indicating VTN conversion into nanofibers. In contrast, only minor diameter variations were detected in VDN solutions (Fig. 3, D and E). The secondary structural transition of VTN peptides during transformation was analyzed by circular dichroism (CD) spectroscopy. After 6 hours of incubation with VCAM-1, VTN exhibited characteristic CD signals with a distinct positive peak at 195 nm and negative peak at 216 nm, demonstrating VCAM-1–induced self-assembly into β sheet–rich nanofibers through hydrogen bonding interactions (Fig. 3F). VDN failed to display typical β sheet signatures, consistent with their nontransformable nature (Fig. 3G). TEM images demonstrated exclusive VCAM-1–triggered nanofiber assembly in the targeted VTN nanoparticles, whereas nontargeted VDN failed to undergo structural reorganization under identical conditions (Fig. 3H). After 24 hours of incubation with recombinant human VCAM-1 (20 μM), VTN self-assembled into unbranched nanofibers with uniform diameters of 10.2 ± 0.8 nm and lengths exceeding 200 nm. Furthermore, CD spectra of VTN showed progressively enhanced β sheet characteristics (intensified positive signal at 195 nm and negative signal at 216 nm) with increasing VTN concentrations. Both VTN and VDN exhibited identical critical micelle concentration (CMC) values of 0.07 mg/ml (Fig. 3I and fig. S9), demonstrating that VTN and VDN acquires enhanced self-assembly capability through precisely increased N-terminal hydrophobicity. To validate the capture of small molecules by VTN and VDN, we monitored the ultraviolet-visible absorption signals with VTN and VDN as the donor and Nile Red (NR) as the acceptor (Fig. 3J and figs. S10 and S11). The VTN and VDN captured and locked one NR molecule in each hydrophobic domain. In the preceding experiments, we confirmed the stable formation of nanofibers, laying the groundwork for their potential antiadhesion effect. To further elucidate the underlying mechanism, we analyzed single-cell transcriptomic data to examine the interactions between immune cells and ECs. The results showed that, under normal conditions, the EC3 subcluster communicated most frequently with immune cells, whereas such interactions were markedly attenuated under xenotransplantation (Fig. 3K). We further investigated the specific ligand-receptor pairs mediating these interactions and identified the VCAM-1–integrin axis as the major pathway. Although this pathway remained detectable under xenotransplantation, its interaction strength was substantially weakened (Fig. 3L). These findings suggest that preserving EC3 integrity while inhibiting immune-endothelial adhesion may represent a critical strategy for regulating transplant immune responses, thereby providing a mechanistic rationale for the subsequent validation of the antiadhesion effect of nanofibers.

### The morphological characterizations on cell surface

**Fig. 4.**
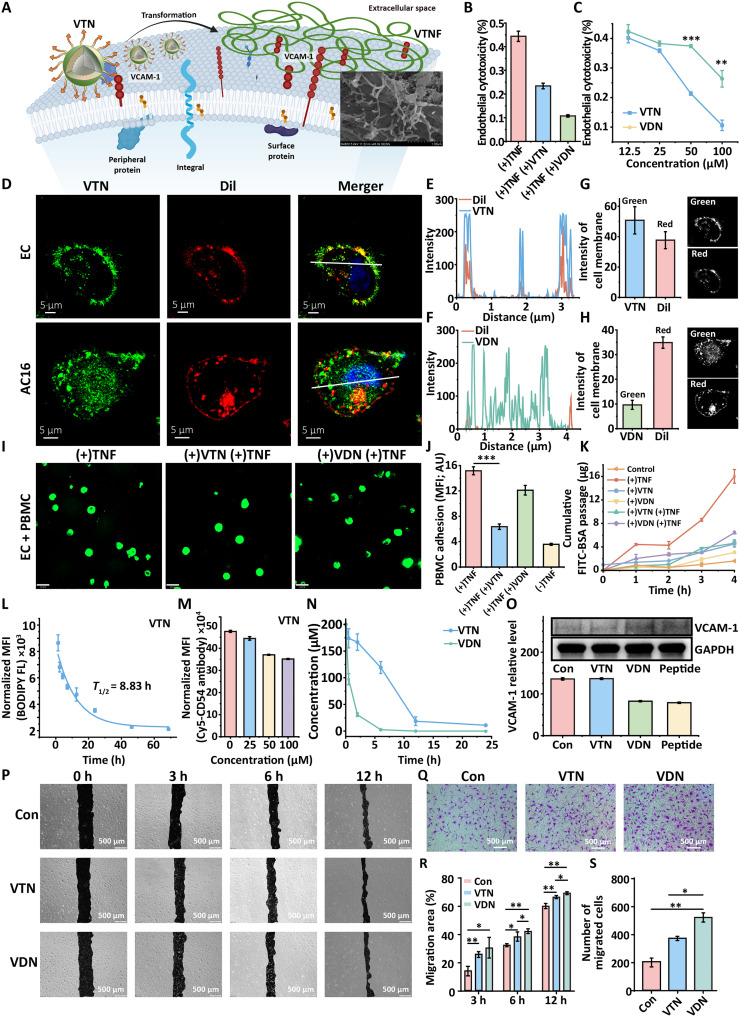
Functional validation of VTN-mediated endothelial protection in vitro. (**A**) Schematic of VCAM-1–triggered VTN transformation into nanofibrous barriers on EC surfaces (insert SEM image from fig. S13). (**B** and **C**) VTN and VDN reduce PBMC-mediated cytotoxicity concentration dependently. (**D** to **H**) Confocal laser scanning microscopy (CLSM) shows specific membrane targeting of VTN on HUVECs versus AC16 cells. (**I** and **J**) Inhibition of PBMC adhesion. BSA, bovine serum albumin; AU, arbitrary unit; MFI, mean fluorescence intensity. (**K**) Preservation of endothelial barrier function under tumor necrosis factor–α (TNF-α) stimulation. (**L**) VTN membrane residence half-life. (**M**) Dose-dependent VCAM-1 epitope blockade. (**N**) Plasma stability. (**O**) Unchanged VCAM-1 protein levels. GAPDH, glyceraldehyde-3-phosphate dehydrogenase. (**P** to **S**) Enhanced HUVEC migration in scratch and Transwell assays. Data are means ± SEM; *P < 0.05, **P < 0.01, and ***P < 0.001 by analysis of variance (ANOVA) or *t* test. h, hours. (A) Created in BioRender. Y. Li, (2026) https://BioRender.com/o2invd9.

Figure 4A shows a schematic diagram of the transformation of VTN on the EC3 surface: Schematic diagram showing the VCAM-1–triggered structural rearrangement of VTN—from nanoparticles to VCAM-1^+^ nanofibrous “barriers” on the EC3 surface, which prevent immune cell adhesion and release of immunosuppressive substances. For functional assessment of VTN-mediated immunoprotection, IL-2–activated peripheral blood mononuclear cells (PBMCs) were cocultured with tumor necrosis factor–α (TNF-α–treated) HUVEC, translating to 68.2 ± 6.3% reduction in PBMC-mediated cytotoxicity via lactate dehydrogenase (LDH) release assays (Fig. 4B). Comparative analysis of VTN and VDN further revealed concentration-dependent enhancement of immunosuppressive activity (Fig. 4C). Membrane-targeting specificity was validated through confocal laser scanning microscopy (CLSM) imaging of VCAM-1–expressing HUVEC, where VTN exhibited precise membrane localization evidenced by strong colocalization with Dil membrane stain (Fig. 4, D and E), concordant fluorescence intensity peaks in line-scan analyses (Fig. 4, F and G), and minimal internalization, unlike its behavior in control AC16 cardiomyocytes (Fig. 4H). VTN and VDN treatments reduced PBMC adhesion by 2.4-fold and 1.3-fold, compared with controls (Fig. 4, I and J), demonstrating inhibition of immune cell–antigen interactions. Before in vitro cell experiments, we conducted cytotoxicity tests on VTN and VDN, and the results indicated that VTN and VDN exhibited >90% cell viability at concentrations ≤200 μM (fig. S12), confirming biocompatibility. To evaluate the integrity of endothelial barriers following VTN and VDN modifications, we measured the permeability of fluorescein isothiocyanate (FITC)–labeled bovine serum albumin across HUVEC monolayers (Fig. 4K). HUVECs were used as an in vitro model on the basis of their well-established relevance to endothelial pathophysiology and conserved VCAM-1–mediated inflammatory responses across species (*28*). TNF-α can increase endothelial permeability and be used as a positive control group. While VTN and VDN treatments exhibited minimal impact on baseline macromolecular permeability, notably, VTN and VDN treatment attenuated TNF-α–induced inflammatory hyperpermeability, suggesting that both VTN and VDN might mitigate TNF-α–mediated barrier dysfunction. Flow cytometric analysis demonstrated a surface residence half-life (*T*_1/2_) of ~8.83 hours for VTN on HUVEC (Fig. 4L). Subsequent investigation of immunoprotective mechanisms demonstrated that VTN achieved dose-dependent blockade of accessible VCAM-1 epitopes on TNF-α–stimulated HUVEC (Fig. 4M). To further evaluate the physicochemical stability and self-assembly behavior of VCAM-1 target peptides, VTN and VDN were incubated in human plasma for different time intervals, and the residual concentrations were quantified by high-performance liquid chromatography (HPLC). Both peptides exhibited good plasma stability, retaining more than 70% of their initial concentration after 24 hours (Fig. 4N). In addition, scanning electron microscopy (SEM) provided direct morphological evidence of nanofiber formation on VTN-treated ECs, as indicated by the presence of distinct fibrous structures compared with the untreated group (fig. S13). The results showed that VTN did not have a significant effect on the expression of VCAM-1 in ECs (Fig. 4O and figs. S14 and S15), laying the foundation for subsequent in vivo experiments. The VTN and VDN fibrous network on ECs is designed to bind VCAM-1 and form nanofibers, which are hypothesized to influence immune cell adhesion. To assess the impact of VTN and VDN on HUVEC migration, wound-healing assays were performed to evaluate angiogenesis-related effects. VTN- and VDN-treated HUVEC demonstrated a significantly larger wound-healing area compared with the control group, suggesting enhanced migratory capacity. These observations indicate that the VCAM-1–binding nanofibers formed by VTN and VDN may promote HUVEC migration during angiogenesis (Fig. 4, P and R). To further evaluate the effect of VTN and VDN on cell migration, a Transwell assay was performed. Results demonstrated that VTN- and VDN-treated HUVEC exhibited a significantly higher migration rate than the control group, as quantified by the number of migrated cells. Moreover, VDN outperformed VTN in promoting migration (*n* = 5, *P* < 0.05, Fig. 4, Q and S).

### Biodistribution and biocompatibility evaluation of VTN and VDN in vivo

**Fig. 5.**
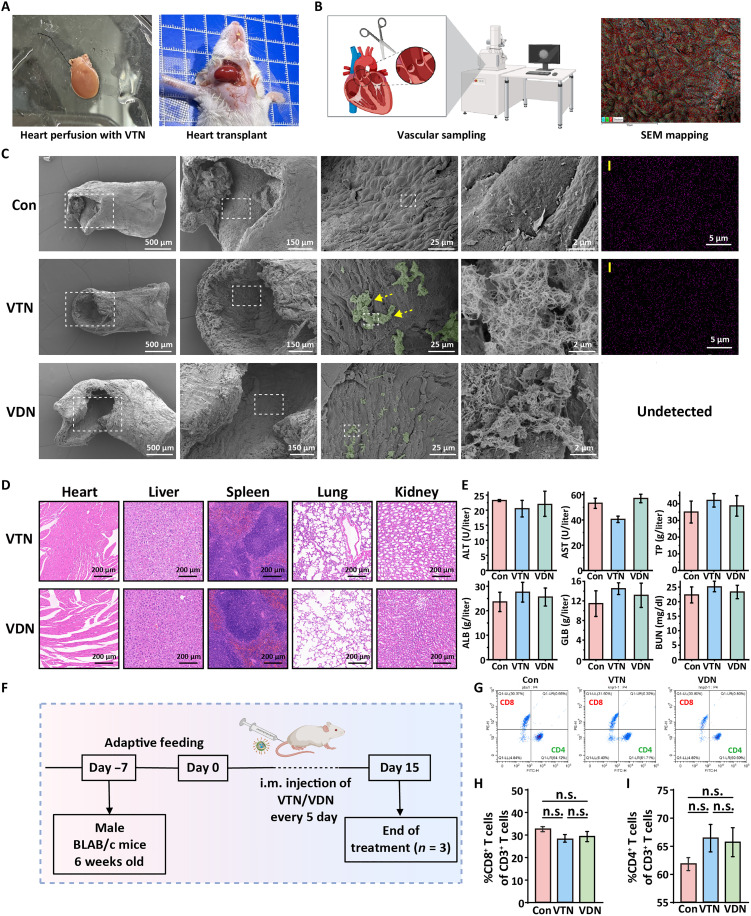
Biodistribution and biocompatibility of VTN and VDN in CXTx. (**A**) Heart perfusion with VTN and transplantation images. (**B**) Vascular sampling schematic and SEM mapping. (**C**) SEM images and elemental mapping of vascular lumens [control (Con), VTN, and VDN; yellow arrows, nanofibers] (a different scale was shown in fig. S20). (**D**) H&E-stained major organs (scale bars, 200 μm). (**E**) Serum biochemistry markers (means ± SEM). ALT, alanine aminotransferase; AST, aspartate aminotransferase; TB, total bilirubin; ALB, albumin; GLB, globulin; BUN, blood urea nitrogen. (**F**) Experimental timeline for intramuscular (i.m.) injections and analysis. (**G**) Representative CD4/CD8 flow cytometry plots. PE-H, phycoerythrin height; FITC-H, fluorescein isothiocyanate height. (**H** and **I**) Quantification of CD8^+^ and CD4^+^ T cells (n.s., not significant). Scale bars as indicated. (B) and (F) Created in BioRender. Y. Li, (2026) https://BioRender.com/o2invd9.

To quantitatively track nanoparticle biodistribution, VTN and VDN were radiolabeled with iodine-127 before perfusion (figs. S16 to S19). The biological distribution of VTN and VDN in grafts was evaluated in vivo. VTN was perfused into the heart during transplantation, and, then, the localization of nanoparticles in cardiac tissue was confirmed by SEM and energy spectrum (Fig. 5, A and B, and fig. S21). SEM imaging revealed that, after 24 hours of VTN and VDN treatment, the luminal surface of donor hearts exhibited extensive deposition of VTN and VDN nanofibers. Higher-magnification analysis demonstrated a well-defined, interconnected nanofiber network. Notably, the VTN group displayed a more uniform and densely distributed fiber morphology compared with VDN, consistent with the physicochemical characterization data of VTN and VDN. Conversely, no fibers were observed in control group (Fig. 5C and fig. S20).

Comprehensive evaluation of VTN biocompatibility demonstrated no treatment-related toxicity across all analyzed physiological systems. Histopathological examination of major organs (liver, kidney, spleen, heart, and lung) revealed intact tissue architecture with no evidence of necrosis, inflammation, or fibrosis compared with untreated controls (Fig. 5D). Furthermore, blood biochemical analysis showed no abnormal fluctuations in liver function markers (alanine aminotransferase, aspartate aminotransferase, total bilirubin, albumin, globulin, and blood urea nitrogen) between the NC, VTN, and VDN groups (Fig. 5E). No abnormal indicators were found in the blood routine test either (table S1), indicating that VTN and VDN neither induces hepatocyte damage nor disrupts protein synthesis. The hemolysis test results showed that no obvious red blood cell hemolysis was observed for VTN and VDN within the tested concentration range, indicating that both nanomaterials have good blood compatibility (fig. S22).

Immunological homeostasis evaluation experimental workflow: Diagram showing the sequence of VTN and VDN delivery, tissue collection, and flow cytometric analysis of cardiac transplant T cell populations to measure immunological disruption (Fig. 5F). Quantitative analysis revealed comparable proportions of both CD4^+^ and CD8^+^ T cell populations in cardiac tissue of VTN and VDN-treated mice relative to NCs (Fig. 5, G to I), indicating that VTN and VDN administration did not induce significant T cell infiltration or alter the helper/cytotoxic T cell balance. These results demonstrate efficient nanoparticle retention and excellent biocompatibility within the graft vasculature, with VTN exhibiting superior structural stability.

### In vivo therapeutic assessment of VTN and VDN

**Fig. 6.**
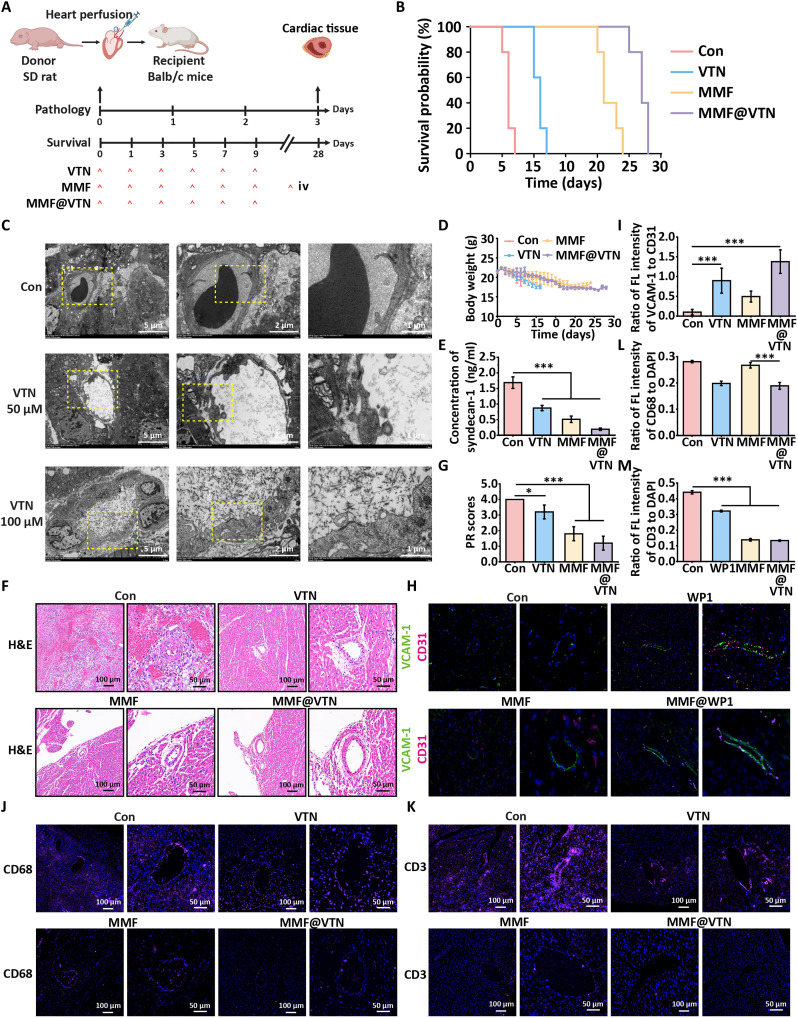
In vivo therapeutic efficacy of VTN in rat-to-mouse CXTx. (**A**) Experimental design: Donor SD rat hearts perfused with VTN/vehicle, transplanted into Balb/c recipients. Four groups (*n* = 5): control (Con), VTN, MMF, and MMF@VTN. Intravenous (iv) dosing every other day; endpoint day 3 or survival. (**B**) Kaplan-Meier survival curves (median: Con, 6.7 days; VTN, 12.1 days; MMF, 21.8 days; MMF@VTN, 27.0 days; log-rank test). (**C**) TEM of graft vasculature showing dose-dependent nanofiber coating on endothelium with VTN (50/100 μM; yellow boxes; scale bars, 5/2/1 μm). (**D**) Recipient body weight over 30 days. (**E**) Serum syndecan-1 levels. (**F**) H&E-stained graft sections (scale bars, 100/50 μm). (**G**) Parenchymal rejection (PR) scores. (**H**) VCAM-1 (green)/CD31 (red) immunofluorescence. (**I**) VCAM-1/CD31 fluorescence ratio. (**J** and **K**) CD68^+^ macrophage (J) and CD3^+^ T cell (K) immunofluorescence (DAPI blue; scale bars, 100/50 μm). (**L** and **M**) Quantification of CD68 and CD3 intensity. Data means ± standard deviation (*n* = 5). *P < 0.05 and ***P < 0.001 by ANOVA or *t* test. (A) Created in BioRender. Y. Li, (2026) https://BioRender.com/o2invd9.

MMF was selected on the basis of biological considerations. MMF effectively inhibits lymphocyte proliferation while exhibiting lower endothelial toxicity than calcineurin inhibitor (CNI) inhibitors, aligning with our endothelium protective strategy. To evaluate the therapeutic efficacy of VTN and VDN in vivo, a cardiac xenografts model was established with four treatment groups (*n* = 5 per group): (i) control (cardiac xenografts control), (ii) VTN (targeting VCAM-1), (iii) MMF (immunosuppressant control), and (iv) MMF@VTN (combination therapy). The donor hearts pretreated with either VTN or control solution via antegrade aortic perfusion were transplanted into recipient mice. Subsequently, the therapeutic agents or phosphate-buffered saline (PBS) were administered via tail vein injection every other day. Grafts were harvested for endpoint analysis on postoperative day 3 (Fig. 6A). Kaplan-Meier analysis revealed that the median graft survival time of VTN monotherapy was longer than that of control group (12.1 versus 6.7 days, *P* < 0.001), whereas MMF@VTN achieved a median survival of 27.0 days, significantly exceeding MMF alone (21.8 days, *P* < 0.001; Fig. 6B). The results indicate that the MMF@VTN combination therapy extends the median survival time 4-fold versus control group (27 versus 6.7 days, *P* < 0.001). This synergistic survival benefit corresponded with direct TEM evidence of VCAM-1–triggered nanofiber formation on graft vasculature (Fig. 6C) and significantly reduced endothelial injury biomarkers: Serum syndecan-1 levels decreased 83% in MMF@VTN versus controls (*P* < 0.0001), outperforming both monotherapies (Fig. 6E and fig. S23 to S25). Critically, MMF@VTN treatment attenuated drug toxicity, maintaining stable body weights (Fig. 6D) and reducing HUVEC cellular cytotoxicity by 50% at 50 μM versus MMF alone (fig. S26), confirming nanoparticle-mediated cytoprotection.

Histopathological evaluation by hematoxylin and eosin (H&E) staining and parenchymal rejection (PR) scoring (table S2) demonstrated that VTN-treated grafts preserved significantly more intact ECs with reduced inflammatory infiltration (PR score, 3.2 ± 0.4). This contrasted with the control group, which exhibited severe EC denudation and leukocyte accumulation (PR score, 4.0 ± 0.0). Notably, the MMF@VTN group achieved near-complete EC preservation (PR score, 1.2 ± 0.4), significantly outperforming MMF monotherapy (PR score, 1.8 ± 0.4; *P* < 0.0001) (Fig. 6, F and G). Immunofluorescence analysis demonstrated that both VTN and MMF monotherapy significantly preserved the structural integrity of CD31^+^VCAM-1^+^ ECs, whereas the MMF@VTN group exhibited optimal EC integrity (Fig. 6, H and I). In contrast, control grafts displayed fragmented CD31^+^ ECs, indicating severe endothelial damage. To further evaluate immune cell infiltration, we performed CD3 and CD68 immunofluorescence staining to quantify T cell (CD3^+^) and macrophage (CD68^+^) infiltration in cardiac xenografts (Fig. 6, J and K). Compared with controls, the VTN, MMF, and MMF@VTN groups significantly reduced posttransplant infiltration of both T cells and macrophages. Notably, the VTN and MMF@VTN groups demonstrated superior efficacy in suppressing macrophage infiltration compared with the MMF groups (Fig. 6, L and M). These results establish that MMF@VTN confers dual protection in CXTx by preserving endothelial integrity while simultaneously suppressing immune cell recruitment, thereby improving graft outcomes. VTN prolonged graft survival to 27 days, a fourfold increase over controls, but ultimate failure occurred because of redundant xenograft rejection pathways. While VTN effectively targeted VCAM-1–mediated endothelial activation, other mechanisms, such as antibody-mediated rejection and chronic vascular lesions, eventually breached this barrier. Nonetheless, achieving 27-day survival with nanoparticle monotherapy in a stringent transplant model represents a significant advancement in extending the xenograft therapeutic window.

## DISCUSSION

CXTx remains challenged by species-incompatible endothelial dysfunction, where disrupted molecular homeostasis accelerates graft rejection through thromboinflammation and immune-mediated EC injury. Our single-cell transcriptomic profiling of GTKO porcine-to-primate xenografts identified a pathologically vulnerable VCAM-1^+^ EC3 subpopulation that undergoes selective depletion during graft injury, providing a therapeutic target for precision intervention. To address this vulnerability, we engineered (VTN), a VCAM-1–targeted system that undergoes ligand-triggered structural transformation into nanoscale barrier networks on inflamed endothelium. This bioinspired engineering strategy achieved dual synergistic protection: (i) physical immunoisolation through nanofibers that reduced T cell adhesion by 73% (*P* < 0.001) and macrophage infiltration and (ii) localized delivery of MMF to potentiate therapeutic efficacy while minimizing systemic exposure. These complementary mechanisms extended xenograft survival to 27.0 days, significantly surpassing MMF monotherapy (21.8 days, *P* < 0.001) through endothelial protection and targeted immunomodulation. Continuous treatment is essential during the early posttransplant high-risk period to preserve endothelial protection. Dosing frequency may be reduced as immune adaptation and conventional immunosuppression stabilize. The impact of varied regimens on long-term graft survival warrants systematic evaluation.

While the current study used a rat-to-mouse model for therapeutic validation due to its high-throughput and cost-effective nature, we acknowledge that the pig-to-nonhuman primate (NHP) model remains the gold standard for assessing clinical scalability. Having successfully identified the target endothelial subtype in the pig-to-NHP model and validated VTN efficacy in rodents, our next step is to evaluate this approach in a pig-to-NHP heterotopic heart transplantation model. These ongoing large animal studies will address critical questions regarding VTN scalability, optimal dosing, and administration regimen.

In conclusion, this bioengineered dual-therapy addresses the short survival dilemma in CXTx by decoupling immunosuppression from EC damage, offering a paradigm shift for CXTx medicine. Given the intensity and complexity of xenogeneic immune responses, successful clinical translation will require multilayered interventions. VTN does not obviate the need for genetic modification; rather, it addresses a distinct facet of rejection, endothelial inflammation and activation, that persists even in multigene edited grafts. The combined application of genetically modified donor organs with VTN endothelial protection, therefore, represents the most promising path forward for achieving long-term xenograft acceptance. Future studies should prioritize evaluating long-term biocompatibility of VTN to develop personalized interventions aimed at optimizing graft longevity while preventing systemic toxicity.

## MATERIALS AND METHODS

### Materials

All reagents and solvents for organic synthesis were obtained from commercially available sources and used without further purification. *O*-(benzotriazol-1-yl)*N*,*N*,*N*,*N*′-tetramethyluronium hexafluorophosphate (HBTU), piperidine, *N*-methylmorpholine (NMM), and trifluoroacetic acid (TFA) were purchased from Sigma-Aldrich Chemical Co. All 9-fluorenyl methoxycarbonyl (Fmoc)–protection amino acids and Wang resins were obtained from GL Biochem (Shanghai) Ltd. The AC16 and HUVEC cell lines were purchased from the cell culture center of the Institute of Basic Medical Sciences, Chinese Academy of Medical Sciences (Beijing, China). AC16-specific culture medium, RPMI 1640 medium, fetal bovine serum (FBS), and Dulbecco’s modified Eagle’s medium (DMEM) were purchased from Gibco (USA). 0.25% Trypsin-EDTA and antibiotic solution (penicillin and streptomycin) were purchased from Invitrogen (Invitrogen, Carlsbad, CA). The Cyanine 7 (Cy7)–labeled cholesterol and LDH Assay Kit was obtained from Solarbio Science & Technology Co. Ltd. (Beijing, China). Culture dishes and plates were purchased from Corning (Corning, New York, USA). All agents not specified were acquired from Sigma-Aldrich unless otherwise noted.

### Heterotopic CXTx surgery from GTKO Bama pigs to macaques

This study was conducted at the Animal Experimental Center of Fuwai Hospital, Chinese Academy of Medical Sciences. The donor pigs were 6- to 8-week-old transgenic GTKO Bama pigs, and the recipients were adult macaques weighing ~10 kg. All animals were housed in pathogen-free facilities and handled in strict compliance with national ethical guidelines for animal welfare and experimental procedures. The study protocol was approved by the Institutional Animal Care and Use Committee of Fuwai Hospital [0108-7-800-ZX(X)-019].

The xenotransplantation was performed following a standard heterotopic abdominal xenotransplantation procedure (*14*). The donor pigs were anesthetized, and a midline incision from the xiphoid to the manubrium was made to access the thoracic cavity. The right atrium, inferior and superior vena cava, and left atrium were transected to interrupt venous return. Cold University of Wisconsin (UW) solution was perfused through an ascending aorta cannula to induce cardiac arrest, and ice slush was poured into the thoracic cavity to protect the heart. The vena cava, left atrium, pulmonary artery, and aorta were carefully dissected, leaving sufficient vascular length for subsequent anastomosis. Once excised, the donor heart was removed, and the open ends of the left atrium and vena cava were sealed to prevent contamination. The recipient macaques were anesthetized, and a midline abdominal incision was made to expose and isolate the infrarenal abdominal aorta and inferior vena cava. After heparin administration, vascular clamps were applied, and arteriotomy and venotomy were performed. The donor aorta was anastomosed to the recipient abdominal aorta in an end-to-side fashion, and the donor pulmonary artery was similarly connected to the recipient inferior vena cava. Once the anastomoses were complete, the vascular clamps were gradually released, and the graft heart was manually decompressed to restore blood flow. The heart resumed beating and recovered sinus rhythm. Postoperative monitoring ensured hemodynamic stability of the graft.

Postsurgery, the recipient macaques were transferred to clean facilities for close observation. Graft survival time and functional status were recorded. To evaluate the endothelial injury phenotypes post-CXTx, no immunosuppressive agents were administered. After the grafts ceased functioning, the recipient macaques were euthanized, and myocardial samples were collected for pathological examination and scRNA-seq analysis.

### Single-cell RNA sequencing

#### Cardiac cell isolation and flow sorting

Tissue samples were excised from pig hearts and placed in ice-cold DMEM medium (Gibco, 11885084). After washing with PBS, myocardial tissues were minced into small pieces and washed again to remove residual blood cells. The tissue fragments were digested in Hanks’ balanced salt solution containing type II collagenase (600 IU/ml; Worthington, 43J14367B) at 37°C for 15 min. The resulting cell suspension was filtered through a 70-μm strainer, and digestion was stopped by adding an equal volume of 10% FBS/DMEM. The suspension was centrifuged at 400*g* for 5 min at 4°C, the supernatant was discarded, and the cell pellet was resuspended in 2% FBS/DMEM. This process was repeated three to five times to purify the cells. Last, the single-cell suspension was treated with red blood cell lysis buffer (Beyotime, C3702) for 10 min, filtered, and resuspended in ice-cold 2% FBS/DMEM.

For flow sorting, 7-aminoactinomycin D (7-AAD; BD Biosciences, 559925) was added to the single-cell suspension at a ratio of 1:20. The cells were analyzed on a FACSAria II flow cytometer (BD Biosciences), and 7AAD-negative live cells were sorted for scRNA-seq analysis.

### 10x Genomics library construction, sequencing, and data analysis

Single-cell suspensions were loaded onto a 10x Genomics platform to generate single-cell Gel Beads-in-Emulsion (GEMs). Libraries were constructed using the Single Cell 5′ Library and Gel Bead Kit (10x Genomics, 1000006) and Chromium Single Cell A Chip Kit (10x Genomics, 120236). Reverse transcription and barcoding of RNA molecules occurred within GEMs. Reverse transcription was performed on a thermal cycler (Bio-Rad, C1000 Touch) with the following program: 53°C for 45 min, 85°C for 5 min, and hold at 4°C. cDNA was purified using DynaBeads Myone Silane Beads and assessed for quality using the Agilent Bioanalyzer High Sensitivity Kit. The libraries were then sequenced on an Illumina HiSeq X-ten system (High Output V2 kit, 150 cycles).

Sequencing data were processed using the 10x Genomics Cell Ranger 6.1.2 pipeline for demultiplexing and alignment, referencing the pig genome (Sscrofa11.1) from Ensembl. The resulting unique molecular identifier (UMI) count matrix was converted into a Seurat (version 3.2.2) object. Quality control included filtering cells with UMI counts below 500 or above 11,000, mitochondrial gene content exceeding 10%, and ribosomal gene content exceeding 20%.

### Sample integration, dimensionality reduction, and clustering

Batch correction of single-cell samples was performed using Seurat’s standard integration workflow, including the Select Integration Features, Find Integration Anchors, and Integrate Data functions. The integrated data were normalized using Scale Data, and the effects of mitochondrial reads, total UMI count, and mitochondrial gene proportion were regressed out before principal components analysis (PCA). PCA was conducted with RunPCA, followed by dimensionality reduction using Uniform Manifold Approximation and Projection (UMAP) via RunUMAP (dims = 1:30). Neighbors were identified using the Find Neighbors function (dims = 1:30), and Leiden clustering was performed with FindClusters at a resolution of 0.4, resulting in 12 clusters. Differentially expressed genes were identified using the Find All Markers function with parameters min.pct = 0.25 and logfc.threshold = 0.25. Cell types were assigned to each cluster on the basis of known marker genes. For ECs, a focused subclustering analysis was conducted. A new neighborhood graph was constructed, and Leiden clustering was performed at resolutions ranging from 0.1 to 1.0 to identify cell types. Marker genes were computed, and cell types exhibiting heterogeneous or low-quality gene expression were excluded from further analysis.

### GO functional enrichment and cell-cell interaction analysis

To explore biological functions, Gene Ontology (GO) enrichment analysis was performed using the “enrichGO” function in the cluster Profiler 4.0 package, with the “org.Hs.eg.db” database as the background. Cell-cell communication networks were analyzed using the CellChat tool, leveraging the PPI.human database to infer intercellular signaling and aggregate communication networks. The communication network and signaling pathways between cell populations were visualized, and differences in cell-cell interactions across different genotypes were compared.

### Preparation of VTN and VDN

VTN and VDN peptide was synthesized manually using standard Fmoc solid-phase peptide chemistry. The peptide chain was assembled de novo via solid-phase synthesis based on standard Fmoc chemistry using Wang resin as the solid support. Anhydrous dimethylformamide (DMF) served as the solvent for all synthesis steps. Fmoc deprotection was performed using a solution of 20% (v/v) piperidine in DMF, applied for 10 min per cycle, and repeated twice. For the coupling steps, HBTU (4 mM) and the respective Fmoc-protected amino acid (4 mM) were dissolved in DMF containing NMM (0.4 mM), with a coupling time of 50 min per residue. Following each coupling reaction, unreacted amine termini were capped using a solution of acetic anhydride (10% v/v) and NMM (10% v/v) in DMF for 15 min. The efficiency of Fmoc deprotection and coupling reactions was qualitatively assessed using the ninhydrin test. All synthesis procedures were carried out in a solid-phase peptide synthesis vessel equipped with a frit. Following chain assembly, the peptide was cleaved from the resin using a cleavage cocktail consisting of 95% TFA, 2.5% water, and 2.5% ethanedithiol (v/v) for 3 hours. TFA was removed by rotary evaporation under reduced pressure to yield a concentrated product. The crude peptide was precipitated by addition to cold anhydrous diethyl ether, collected by centrifugation, and dried under vacuum. The crude peptide was purified by preparative reversed-phase HPLC on an Inertsil ODS-3 C18 column (5 μm, 20 mm by 250 mm). Separation was achieved using a linear gradient of acetonitrile/water (both containing 0.1% TFA) as the mobile phase, starting from 5/95 (v/v) and increasing to 70/30 (v/v) over 18 min, followed by a further increase to 90/10 (v/v) over 4 min. The system was maintained at 90/10 (v/v) for 1 min before returning to the initial conditions of 5/95 (v/v) over 3 min.

### Cell culture

HUVECs and AC16 human cardiomyocyte cell line (AC16) were obtained from the Chinese Academy of Science Cell Bank for Type Culture Collection. HUVEC and AC16 cells were cultured in high-glucose DMEM supplemented with 10% FBS and incubated at 37°C under 5% CO_2_.

For the TNF-α–induced HUVEC inflammation assay, HUVEC were stimulated with TNF-α (80 ng/ml) for 4 hours at 37°C. 4′,6-Diamidino-2-phenylindole (DAPI) and Dil stained the nucleus and cell membranes, respectively. Immunofluorescence images were taken by CLSM (Olympus FV3000). Conditions: excitation wavelength: 405 nm for DAPI, 488 nm for FITC-labeled VTN and VDN, and 561 nm for Dil; emission filter: 430 to 470 nm for DAPI, 500 to 540 nm for FITC-labeled VTN and VDN, and 550 to 650 nm for Dil.

### In vitro study of VTN and VDN

VTN and VDN solution (50 μM) was prepared for characterization. Hydrodynamic size analysis was performed via DLS (Zetasizer NanoZS). Morphological transitions were characterized by TEM; NS solution was drop cast onto carbon-coated copper grids, and, after 5 min of contact, excess solution was removed with filter paper. TEM samples were stained with uranyl acetate for 1 min and dried under vacuum before observation. For CLSM (Zeiss LSM710), HUVEC and MCF-7 cells were incubated overnight in complete DMEM medium at 37°C under 5% CO_2_, treated with Cy5-labeled VTN and VDN solution (200 μM, 50 μl), washed with PBS after designated intervals, and imaged in PBS using a 60× objective; all microscopy parameters remained consistent to compare VTN and VDN binding affinity. Cell viability was assessed using the Cell Counting Kit 8 (CCK-8) assay: Cells were seeded uniformly in 96-well plates (100 μl per well), including blank (medium only), negative control (untreated cells), and experimental groups (cells treated with varying VTN and VDN concentrations). Following preincubation for adherence, test compounds were added for 24 hours; subsequently, 10 μl of CCK-8 reagent was added per well, gently mixed, incubated in the dark for 1 hour (time optimized on the basis of metabolic activity), and absorbance of the developed formazan dye was measured at 450 nm (reference wavelength, 600 to 650 nm) using a microplate reader. All procedures were performed aseptically, protected from light, minimizing bubbles and ensuring uniform cell distribution. For H&E staining, tissue sections underwent deparaffinization, rehydration, antigen retrieval, staining (hematoxylin for nuclei and eosin for cytoplasm/extracellular matrix), dehydration, and mounting; stained sections were analyzed using optical microscopy and software (e.g., ImageJ Fiji).

### Rat-to-mouse CXTx surgery

Xenotransplantation was chosen as the experimental model for two reasons: (i) its translational relevance to addressing organ shortages and (ii) its unique pathological features that provide an optimal context for evaluating VTN. Unlike allogeneic rejection, which is predominantly T cell–mediated with relatively gradual onset, xenograft rejection involves explosive innate immune activation that causes rapid, severe endothelial dysfunction and marked VCAM-1 up-regulation. NHP single-cell sequencing data confirmed that selective depletion of the VCAM-1^+^ endothelial subpopulation is a hallmark of xenograft failure, and the resulting VCAM-1–overexpressing endothelial microenvironment provided an ideal setting to validate VTN’s disease-triggered assembly and therapeutic efficacy.

The small animal experimental protocol was approved by the Animal Care and Use Committee of Fuwai Hospital, Chinese Academy of Medical Sciences [approval no. 0108-1-31-ZX(X)-20]. A heterotopic heart transplantation model was established using 2-week-old Sprague-Dawley (SD) rat pups as donors and 6- to 8-week-old BALB/c mice as recipients (*29*–*33*).

In brief, recipient BALB/c mice were anesthetized, and their neck fur was shaved and disinfected with 5% iodine. A midline incision ~2 cm in length was made on the right side of the neck, 1 cm lateral to the midline. Fat and muscle were separated to expose the external jugular vein and carotid artery. The vessels were ligated with 8-0 silk sutures and transected between the ligatures. Vascular cuffs were placed on the severed ends of the vein and artery, secured with microvascular clamps. The vessels were everted over the cuffs using microsurgical forceps and fixed with 8-0 silk sutures. The donor hearts were harvested from anesthetized SD rat pups. A midline abdominal incision was made, and the diaphragm was cut to expose the heart. The thoracic wall was cut along the anterior axillary lines up to the sternoclavicular joints, and the retracted chest wall was secured with vascular clamps. Cold saline was dripped onto the heart to prevent ischemic injury. After thymectomy, the abdominal aorta was severed for exsanguination, and 1 ml of heparinized saline was perfused through the inferior vena cava to flush the heart. The aorta and pulmonary artery were dissected, and remaining vessels were ligated. The donor heart was excised, trimmed, and positioned in the recipient’s neck. The donor aorta and pulmonary artery were looped with lose 8-0 silk sutures. The donor aorta was connected to the recipient carotid artery, and the donor pulmonary artery was connected to the recipient external jugular vein. The sutures were tightened and secured with multiple knots. Upon releasing the vascular clamps on the recipient carotid artery and jugular vein, the donor heart resumed beating within 10 s and turned pink. The neck incision was closed with 4-0 surgical sutures and disinfected.

Postoperatively, the mice were placed on a warm pad to maintain body temperature until they recovered from anesthesia. Once fully awake, they were housed individually with free access to food and water. Graft survival was monitored daily through visual inspection and palpation.

### Histological examination

Heart tissues of mice and human samples were collected and fixed overnight in 4% (v/v) formalin solution, embedded in paraffin, and cut into 5 μm-thick sections. The sections were then subjected to H&E, immunohistochemical, and immunohistochemical fluorescence staining for histopathological evaluations. The H&E and immunohistochemical images were examined by digital microscopy. The fluorescent images were viewed under a stereomicroscope (Leica DMi8, Leica Microsystems, Germany) and quantified by ImageJ. Primary antibodies including CD31 (ab28364) and VCAM-1 (ab134047) were purchased from Abcam, and secondary antibody (A32733) was purchased from Thermo Fisher Scientific.

### Experimental design and blinding

To ensure unbiased assessment, recipient mice were randomly assigned to four experimental groups (control, VTN, MMF, and MMF@VTN) using a computer-generated randomization list. Allocation concealment was maintained by using coded vials for all treatments, prepared by a researcher not involved in the surgical procedures or outcome assessments. All subsequent analyses, including histopathological scoring, immunohistochemistry quantification, and graft survival evaluation, were performed by at least two independent investigators who were blinded to the group allocation. Preestablished inclusion criteria required all recipients to be male BALB/c mice aged 6 to 8 weeks and donors to be SD rat pups weight 20 g. Animals were excluded from analysis only in cases of surgical failure (e.g., intraoperative death) or postoperative complications not related to rejection (e.g., infection), as defined in the study protocol before the experiment.

### Experimental replication

For in vitro studies, all experiments were performed in three independent biological replicates. Each biological replicate consisted of cells from different passages or separate culture preparations. Within each independent experiment, technical replicates (performed in triplicate, e.g., three wells per condition) were used to ensure measurement precision; the replicate values were averaged before statistical analysis to avoid pseudoreplication.

For in vivo studies, sample sizes (*n* ≥ 4 per group) are provided in the figure legends and represent biological replicates. The exact number of independent experiments is also indicated in the figure legend.

### Statistical analysis and visualization

All data are presented as means ± standard deviation. Before analysis, data were tested for normality and homogeneity of variance. Nonnormally distributed data were subjected to further processing or nonparametric tests. For normally distributed data with equal variances, Student’s *t* test was used for comparisons between two groups, whereas one-way analysis of variance (ANOVA) followed by Tukey’s post hoc test was applied for multiple group comparisons. For data with normal distribution but unequal variances, the Tamhane’s T2 method was used. The survival rates of cardiac grafts were analyzed using Kaplan-Meier survival curves. All statistical analyses and visualizations were performed using GraphPad Prism 9.0, with a significance threshold set at *P* < 0.05. Final figure assembly was completed using Adobe Illustrator.
