# Supplementary material for: VCAM-1–targeting peptide assemblies protect vascular endothelium and prolong cardiac xenograft survival
Source: Sci Adv. 2026 Jul 8;12(28):eaec5707. doi: 10.1126/sciadv.aec5707 (PMC13344358; doi:10.1126/sciadv.aec5707)
Supplement: Supplementary file 1 — Figs. S1 to S26 Tables S1 and S2 [file sciadv.aec5707_sm.pdf]

Science Advances
AAAS

Supplementary Materials for

VCAM-1–targeting peptide assemblies protect vascular endothelium and prolong cardiac xenograft survival

Yi-Jing Li *et al.*

Corresponding author: Jiangping Song, fwsongjiangping@126.com

Sci. Adv. **12**, eaec5707 (2026)
DOI: 10.1126/sciadv.aec5707

This PDF file includes:

Figs. S1 to S26
Tables S1 and S2

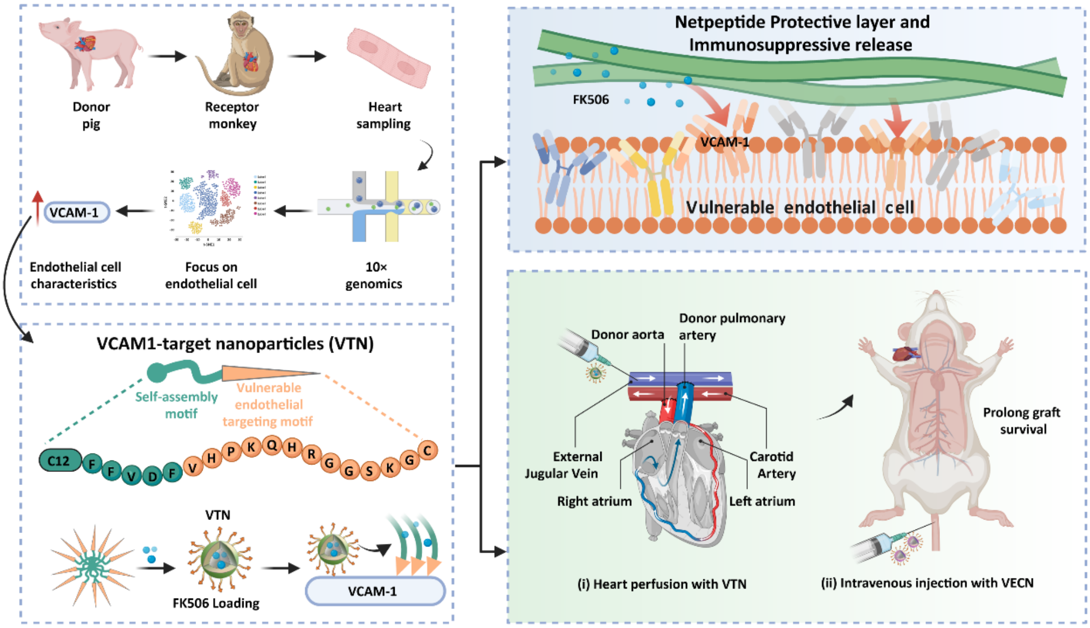

Figure S1. Schematic of VCAM1-Target Nanoparticle (VTN) Nanomaterial System for VCAM1-Targeted Endothelial Protection in Cardiac Xenotransplantation.

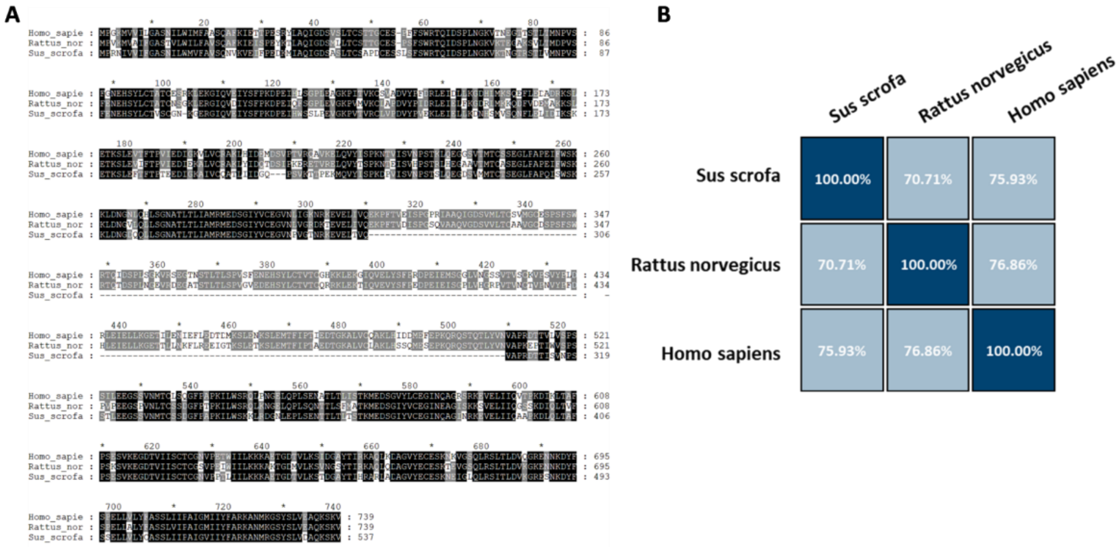

Figure S2. Homology Analysis of the VCAM1 Protein Sequence Across Species. (A) Multiple sequence alignment of VCAM1: Amino acid sequence alignment of VCAM1 from three species: Sus scrofa (UniProt ID: F1S567, porcine), Homo sapiens (UniProt ID: P19320, human), and Rattus norvegicus (UniProt ID: P29534, rat). Sequences were retrieved from the UniProt database (https://www.uniprot.org/). Conserved residues are highlighted in dark blue, and partially conserved residues in light blue—demonstrating high sequence similarity across species, particularly in

functional domains (e.g., immunoglobulin-like repeats critical for ligand binding). (B) VCAM1 sequence identity matrix: Percent identity matrix showing the degree of VCAM1 sequence conservation among Mus musculus (mouse), Homo sapiens (human), and Sus scrofa (porcine). All pairwise comparisons exhibit >70% sequence identity, confirming cross-species conservation of VCAM1. This supports the translational relevance of using human (HUVEC) and rodent (rat-to-mouse) models to evaluate VCAM1-targeted therapeutics for porcine-to-primate cardiac xenotransplantation.

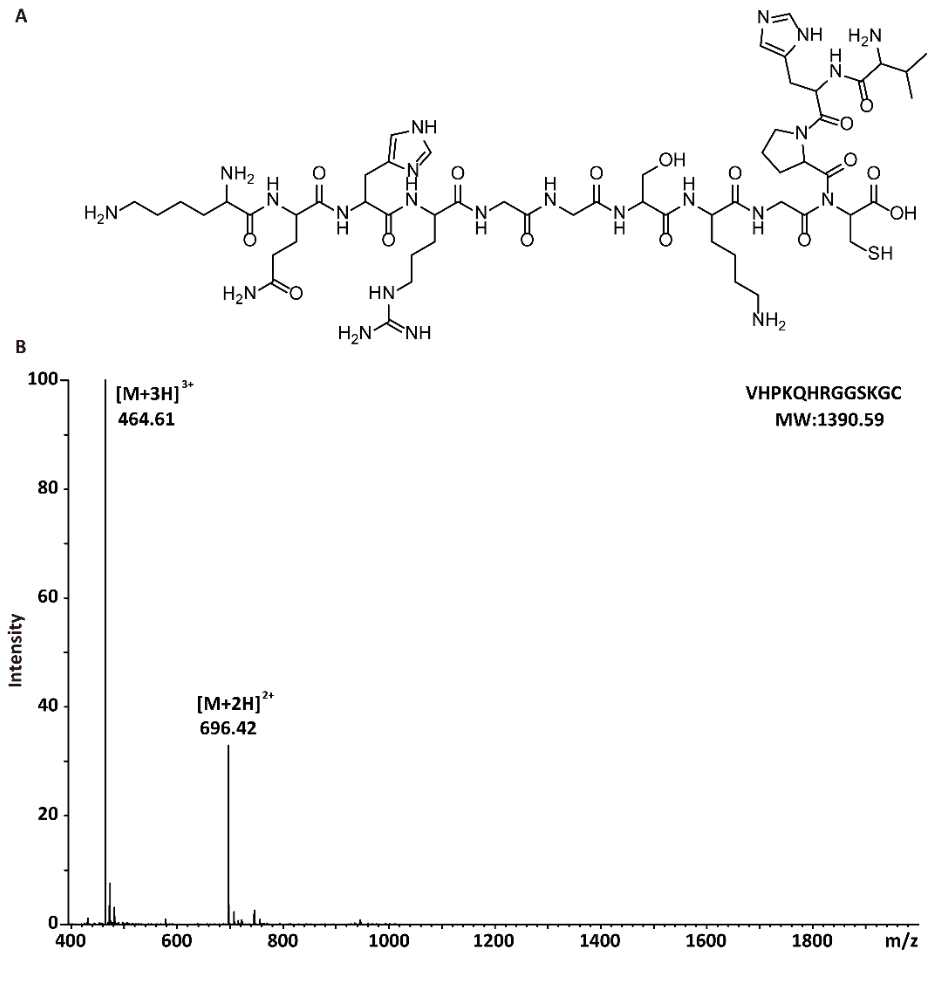

Figure S3. (A) Structural formula and (B) MALDI-TOF mass spectrum of VCAM1-Targeting Peptide: VHPKQHRGGSKGC.

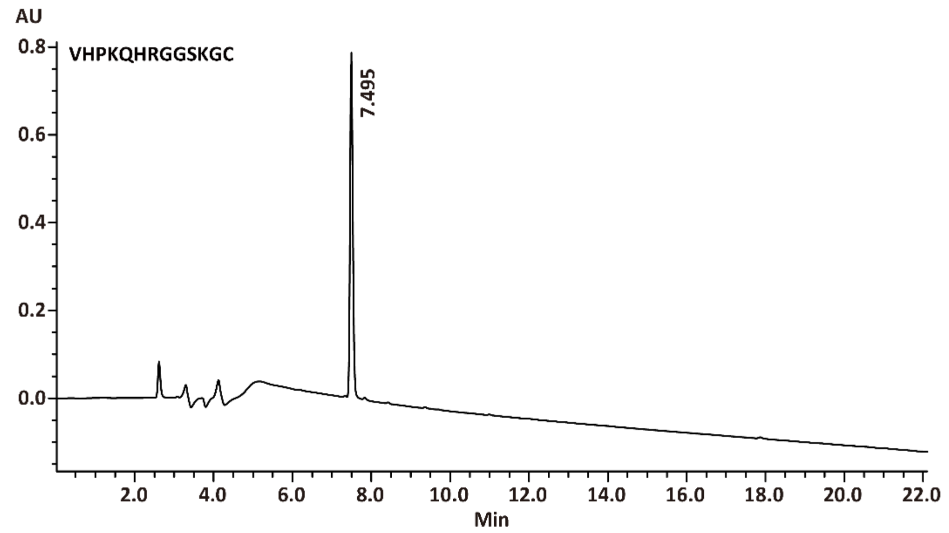

Figure S4. HPLC trace of pure VCAM1 target peptide VHPKQHRGGSKGC.

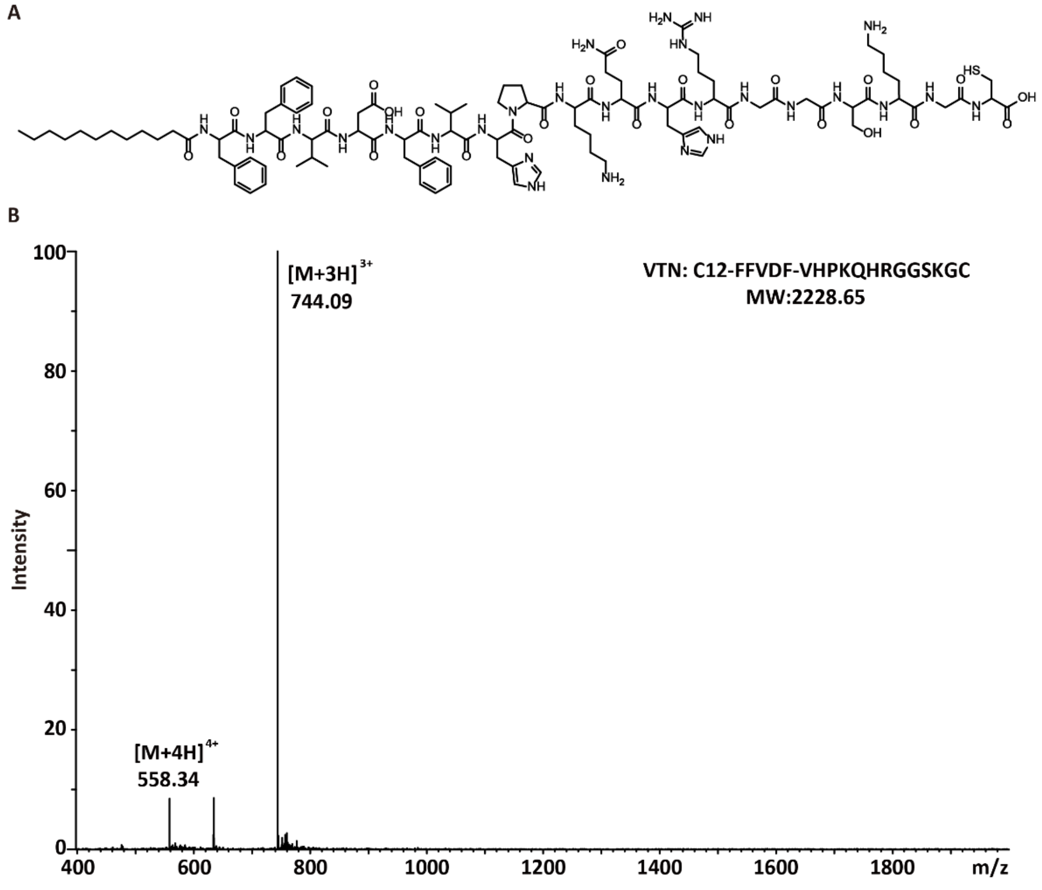

Figure S5. (A) Structural formula and (B) MALDI-TOF mass spectrum of VTN: C12-FFVDF-VHPKQHRGGSKGC.

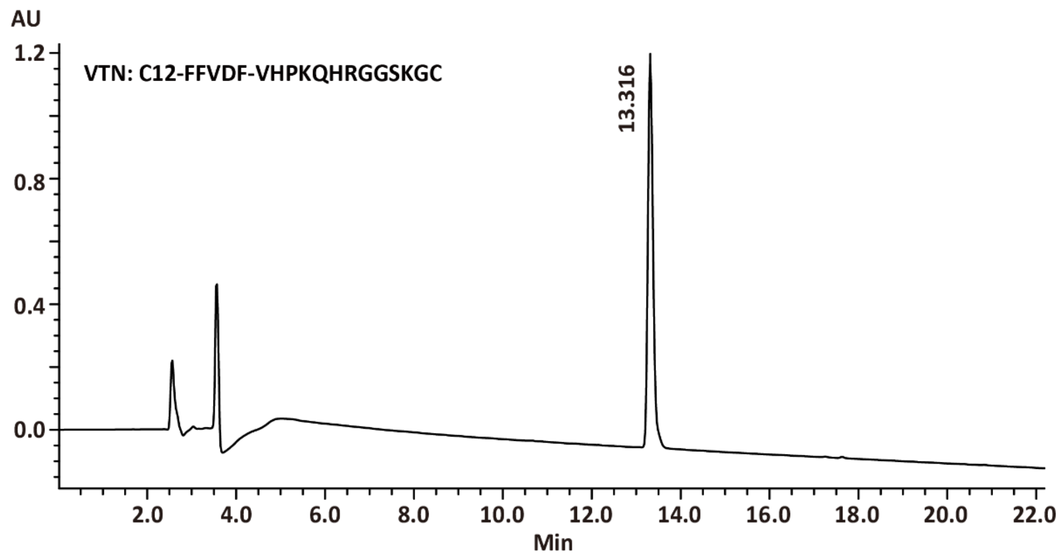

Figure S6. HPLC trace of VTN: C12-FFVDF-VHPKQHRGGSKGC.

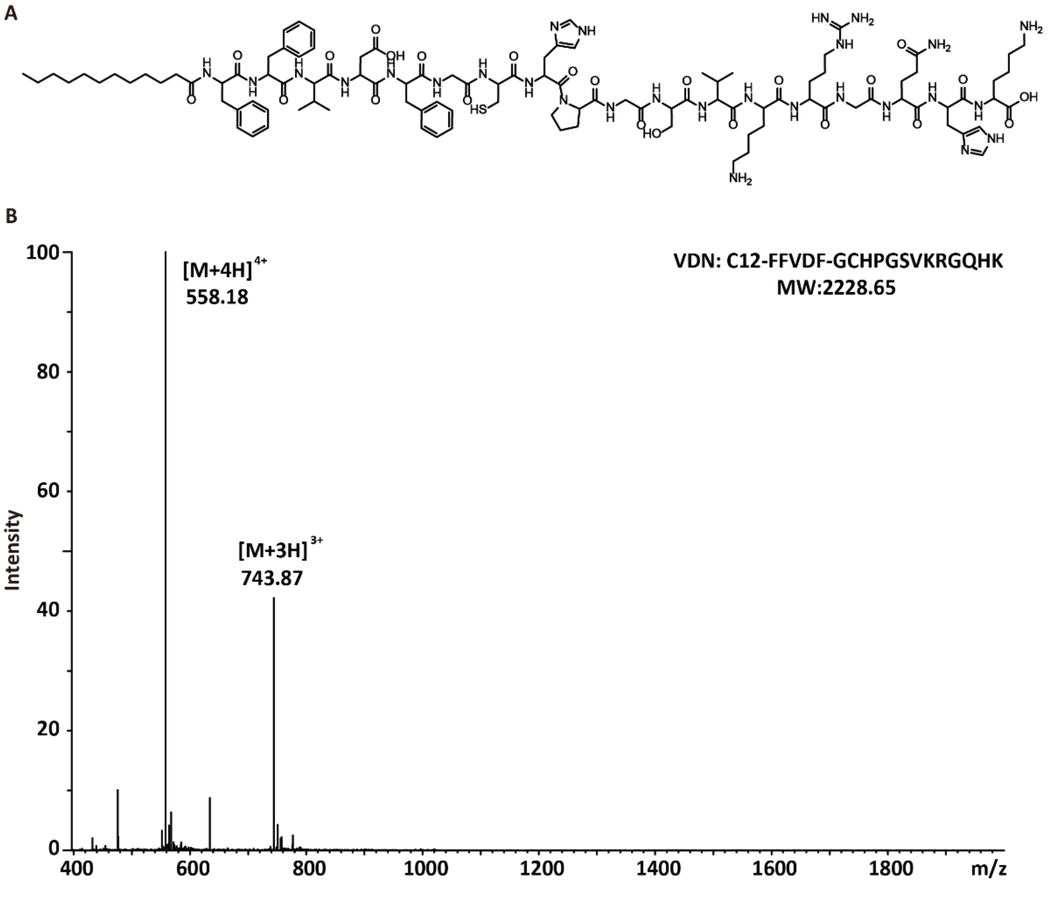

Figure S7. (A) Structural formula and (B) MALDI-TOF mass spectrum of VDN: C12-FFVDF-GCHPGSVKRGQHK.

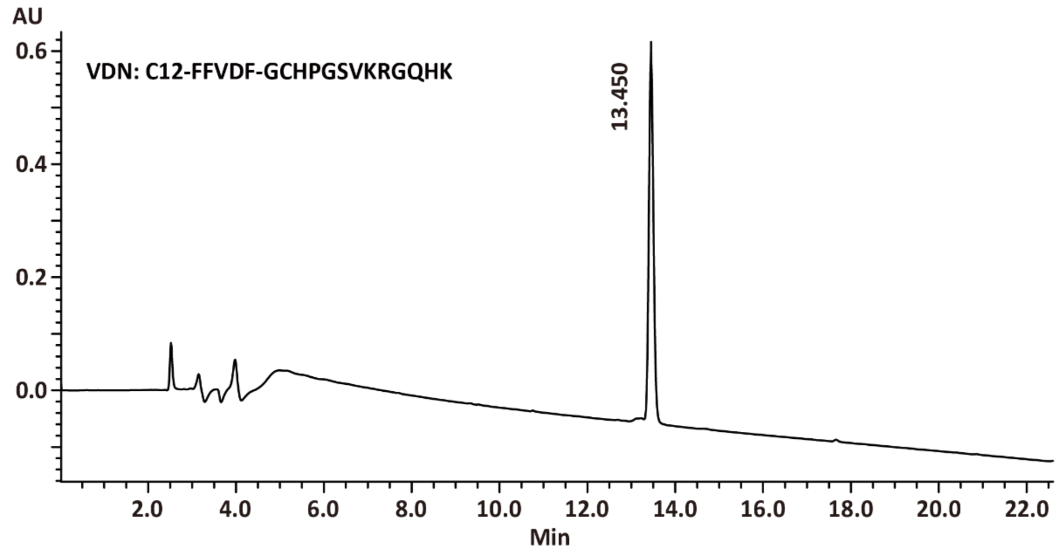

Figure. S8. HPLC trace of VDN: C12-FFVDF-GCHPGSVKRGQHK.

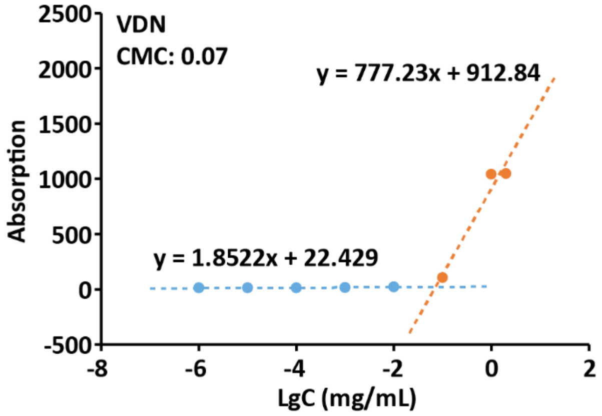

Figure S9. The critical micelle concentration (CMC) of VDN.

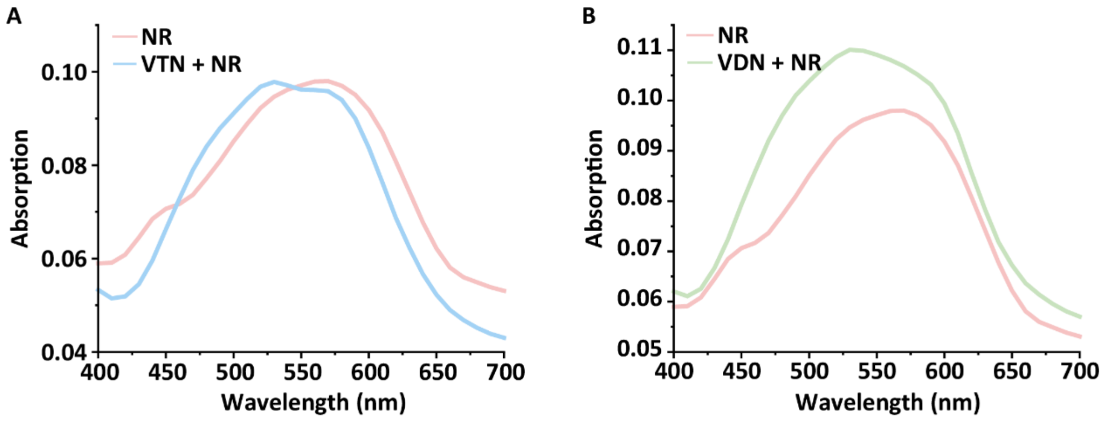

Figure S10. (A) UV-Vis spectra of free Nile Red dye molecules and Nile Red-labeled VTN molecules. (B) UV-Vis spectra of free Nile Red dye molecules and Nile Red-labeled VDN molecules.

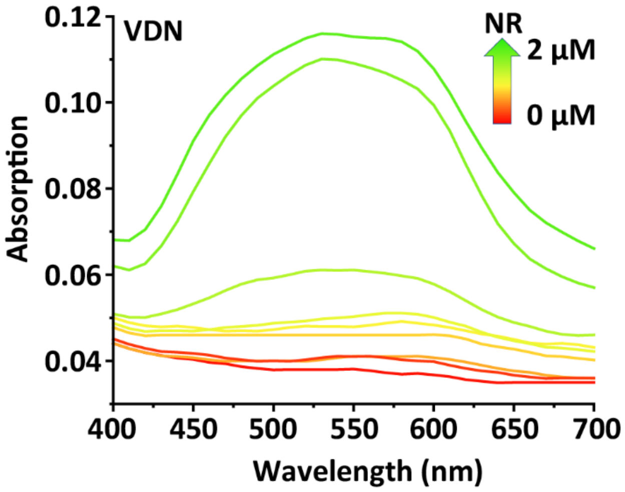

Figure S11. Dependence of UV-Vis absorption spectra on the concentration of Nile Red dye.

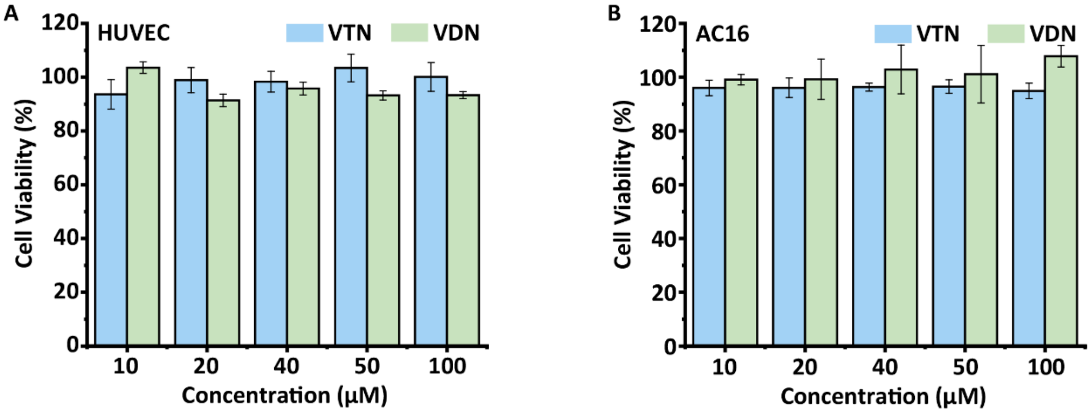

Figure S12. Effects of varying concentrations of VTN and VDN on cellular viability in (A) HUVEC and (B) AC16.

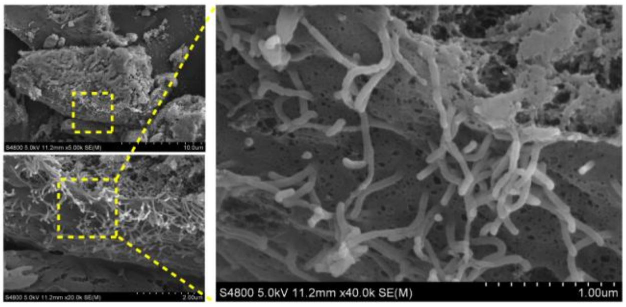

Figure S13. Scanning electron microscopy (SEM) images of VTN-induced nanofiber formation on the surface of endothelial cells. The representative SEM image shows the surface morphology of HUVECs after VTN treatment.

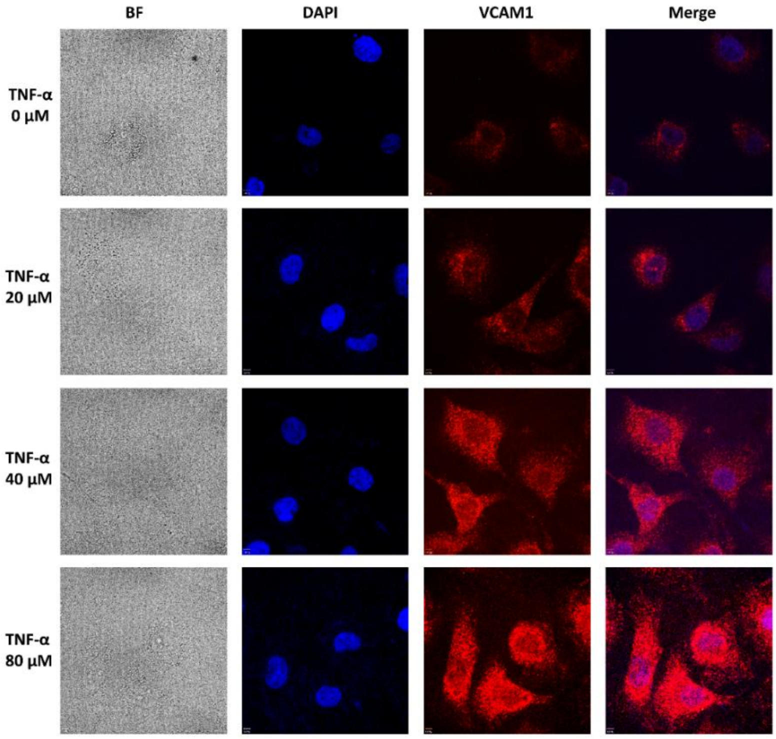

Figure S14. TNF-α Induces VCAM1 Expression in Endothelial Cells in a Dose-Dependent Manner. Immunofluorescence images of endothelial cells (HUVEC) treated with increasing concentrations of tumor necrosis factor-α (TNF-α: 0 μM, 20 μM, 40 μM, 80 μM) to evaluate VCAM1 expression. Each row corresponds to a single TNF-α concentration, with four columns: Bright-field (BF): Phase-contrast images showing cell morphology (no treatment-related cytotoxicity observed). DAPI: Nuclear staining (blue) to visualize cell nuclei and confirm uniform cell density across groups. VCAM1: Immunofluorescence staining (red) targeting VCAM1, with signal intensity reflecting VCAM1 expression levels. Merged: Overlay of DAPI and VCAM1 signals to localize VCAM1 to the cell membrane. Progressive increases in TNF-α concentration correlate with significantly stronger VCAM1 red fluorescence—demonstrating that TNF-α upregulates VCAM1 expression in a dose-dependent manner. This validates the use of 80 μM TNF-α (maximal VCAM1 induction) for subsequent in vitro experiments evaluating VTN-mediated VCAM1 targeting.

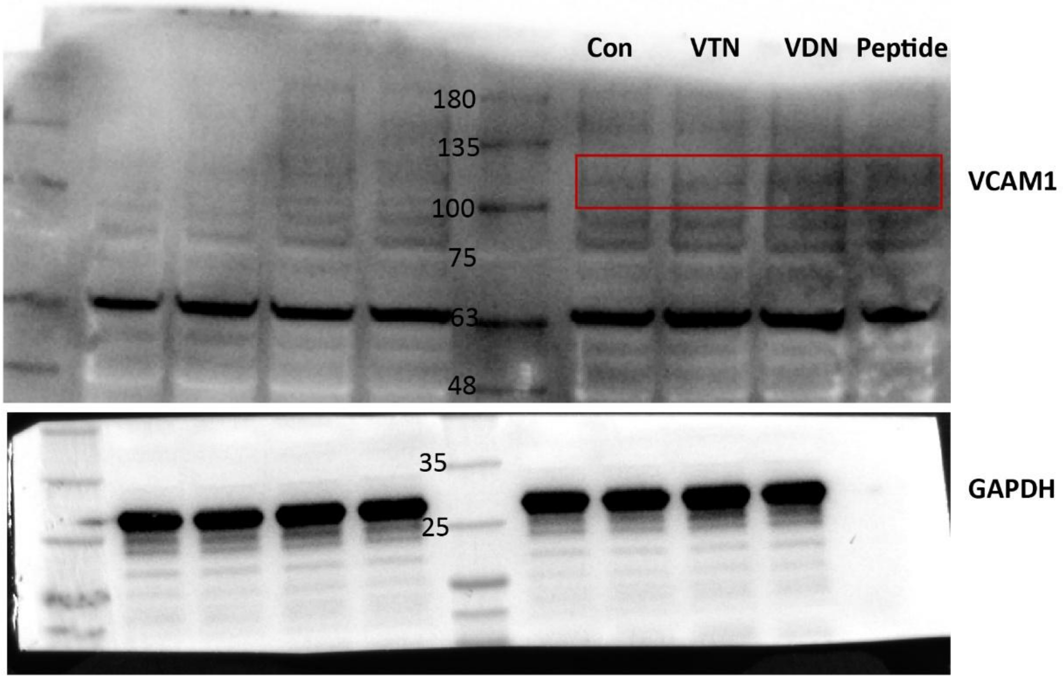

Figure S15. The Western blot results for VTN and VDN.

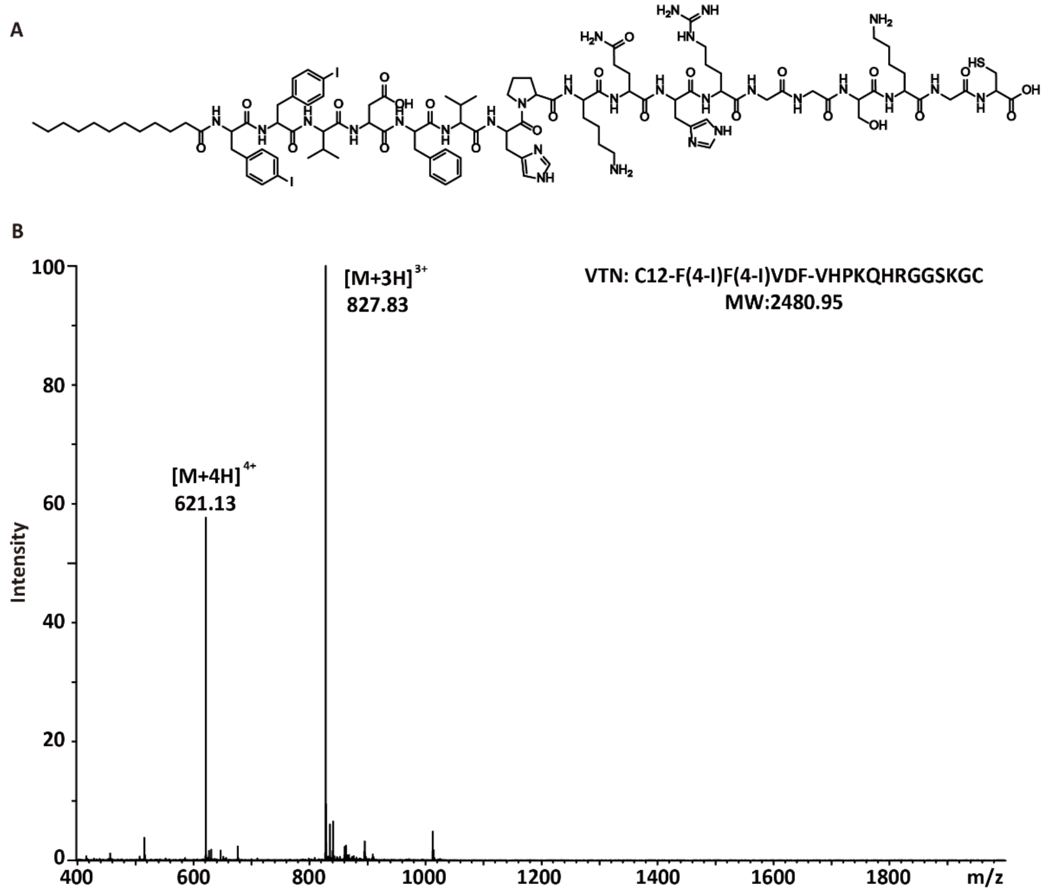

Figure S16. (A) Structural formula and (B) MALDI-TOF mass spectrum of iodine VTN: C12-F(4-I)F(4-I)VDF-VHPKQHRGGSKGC.

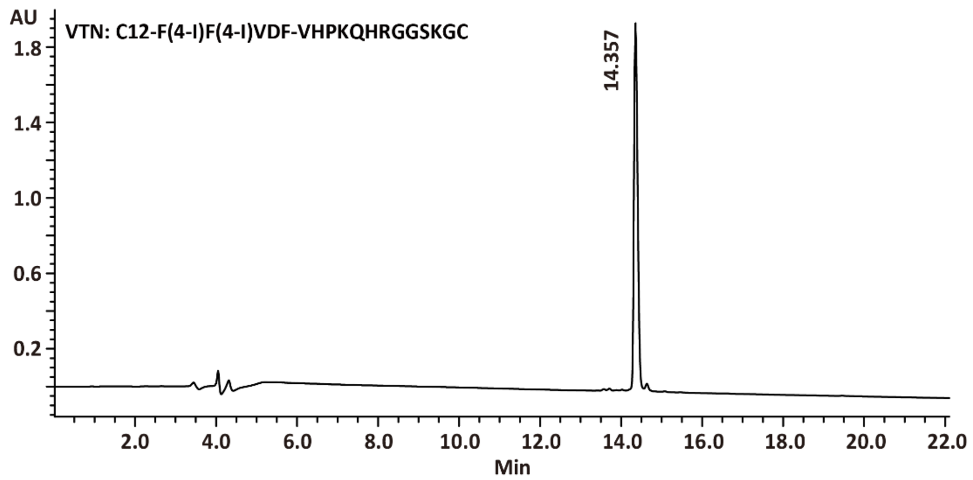

Figure S17. HPLC trace of iodine VTN: C12-F(4-I)F(4-I)VDF-VHPKQHRGGSKGC.

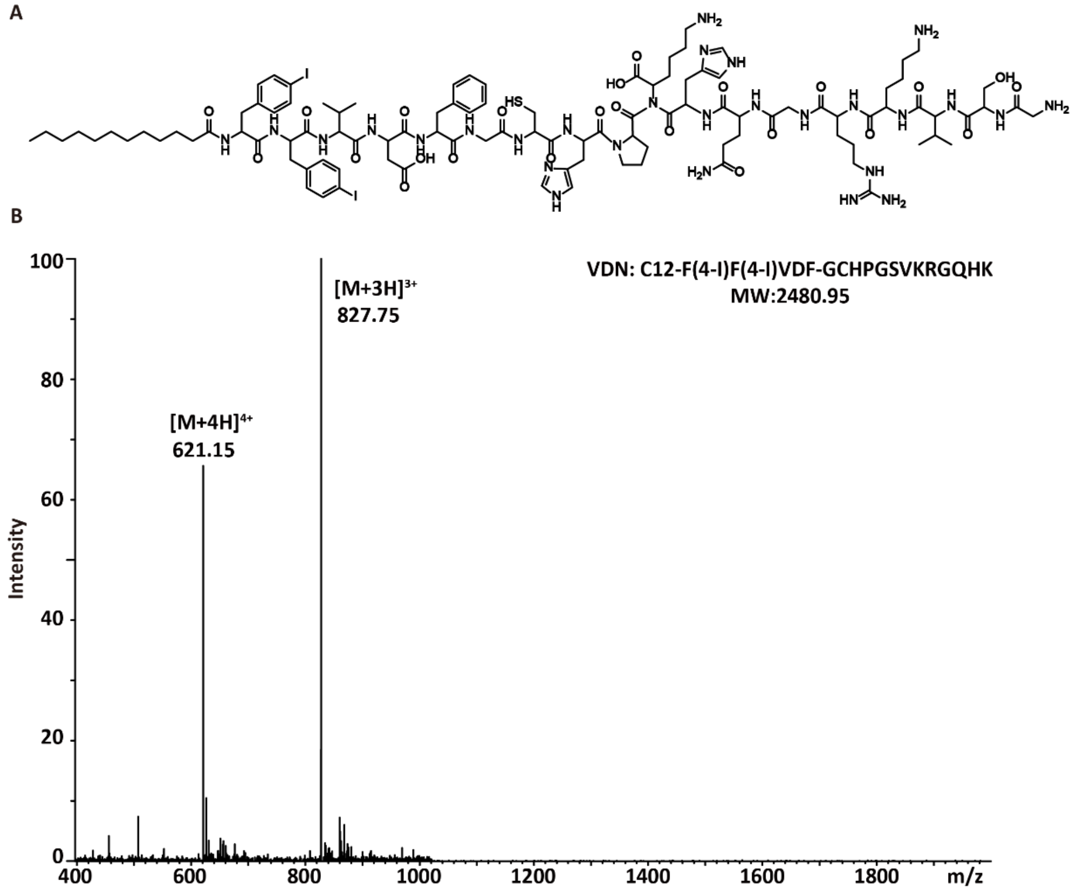

Figure S18. (A) Structural formula and (B) MALDI-TOF mass spectrum of iodine VDN: C12-F(4-I)F(4-I)VDF-GCHPGSVKRGQHK.

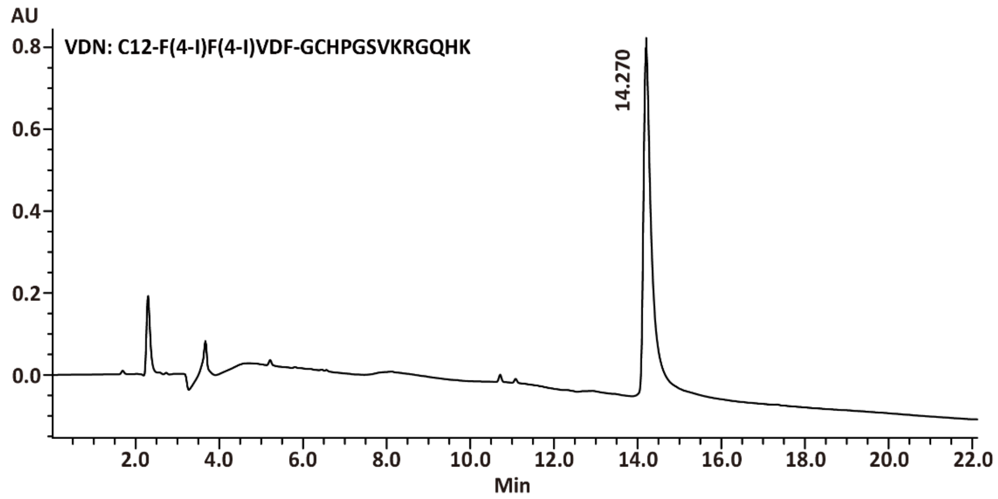

Figure S19. HPLC trace of iodine VDN: C12-F(4-I)F(4-I)VDF-GCHPGSVKRGQHK.

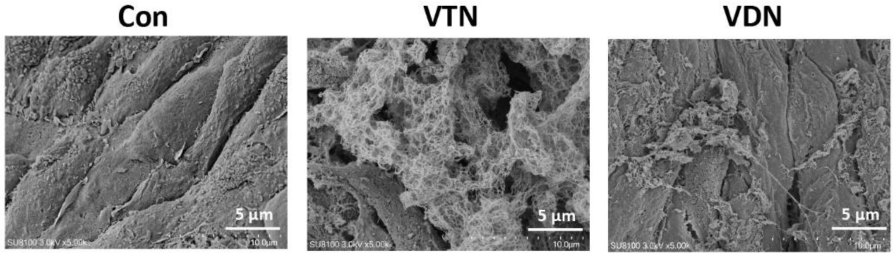

Figure S20. SEM images of VTN and VDN, clearly demonstrating nanofiber architectures on suckling SD rat aorta.

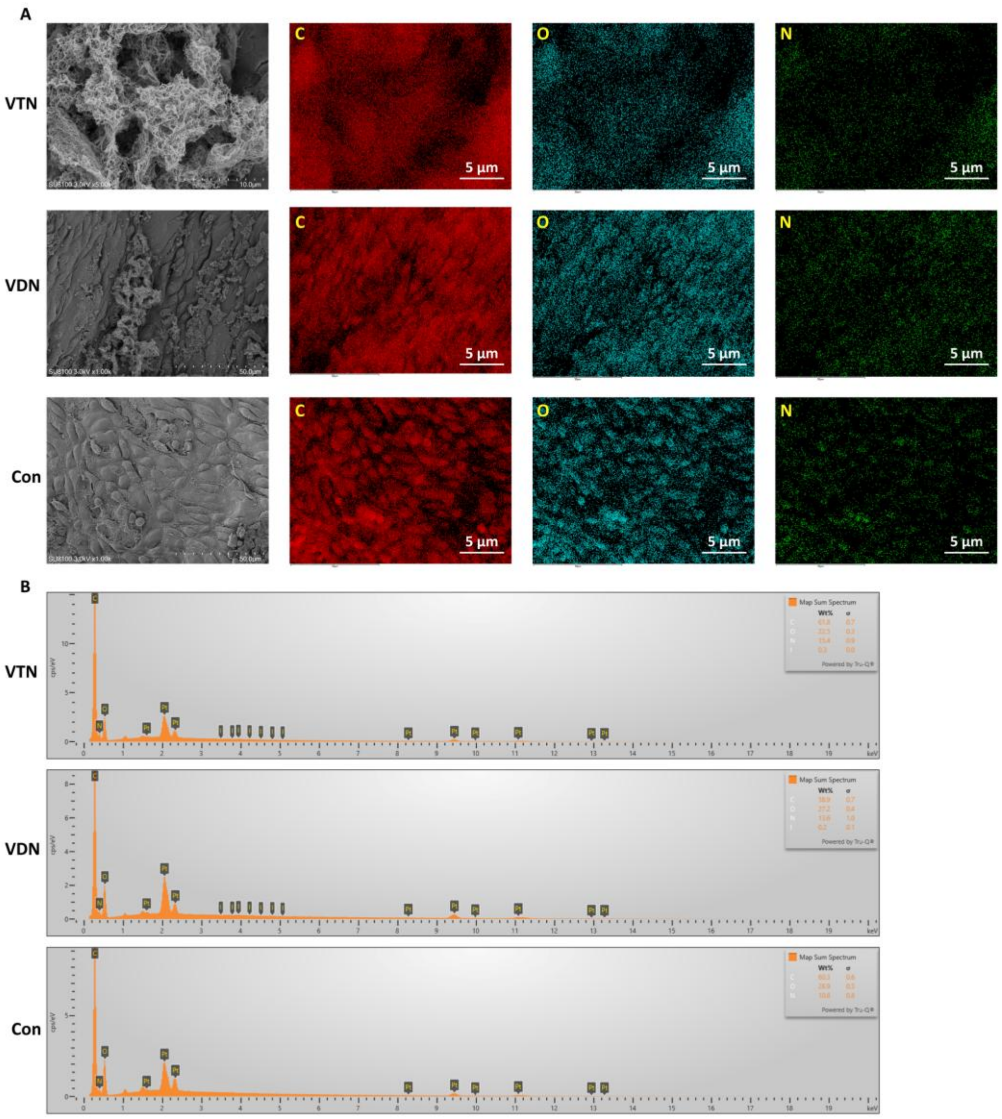

Figure S21. (A) SEM mapping results for VTN, VDN, and control group. (B) Elemental content analysis for VTN, VDN, and control group.

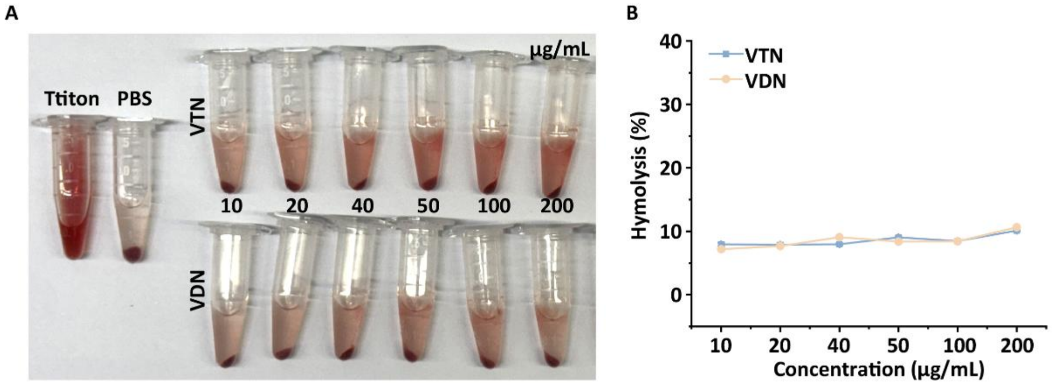

Figure S22. The Effects of VTN and VDN on Erythrocytes. (A) Schematic illustration of hemolytic effects. (B) Influence of VTN and VDN concentrations on hemolytic activity.

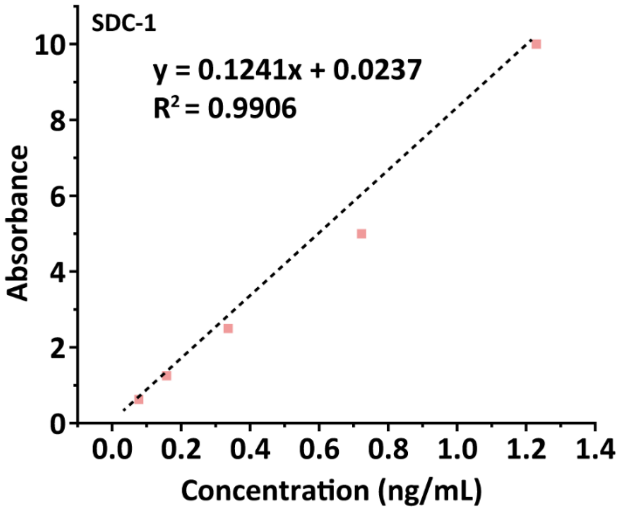

Figure S23. The standard curve for syndecan-1 (SDC1).

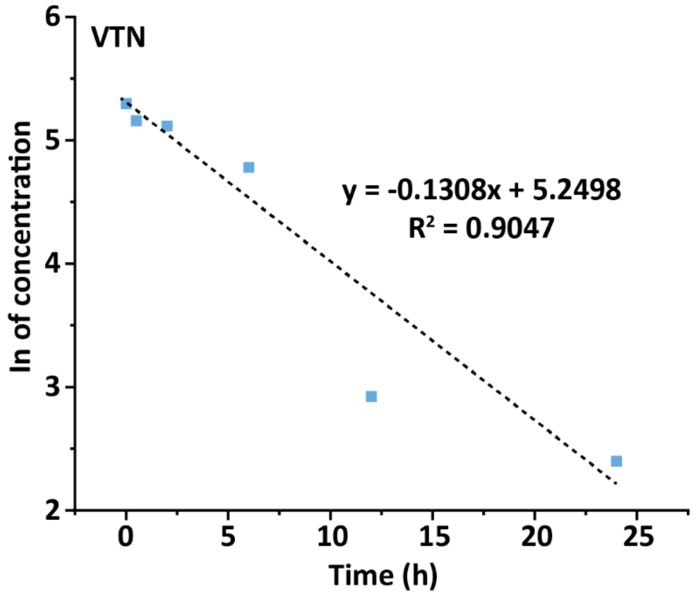

Figure S24. The concentration-time curve of SDC-1 in the VTN group. The vertical axis is displayed on a logarithmic scale.

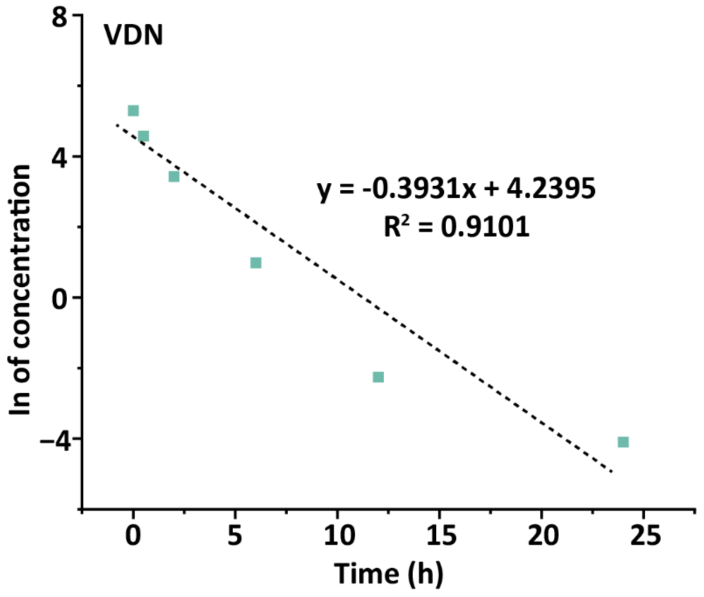

Figure S25. The concentration-time curve of SDC-1 in the VDN group. The vertical axis is displayed on a logarithmic scale.

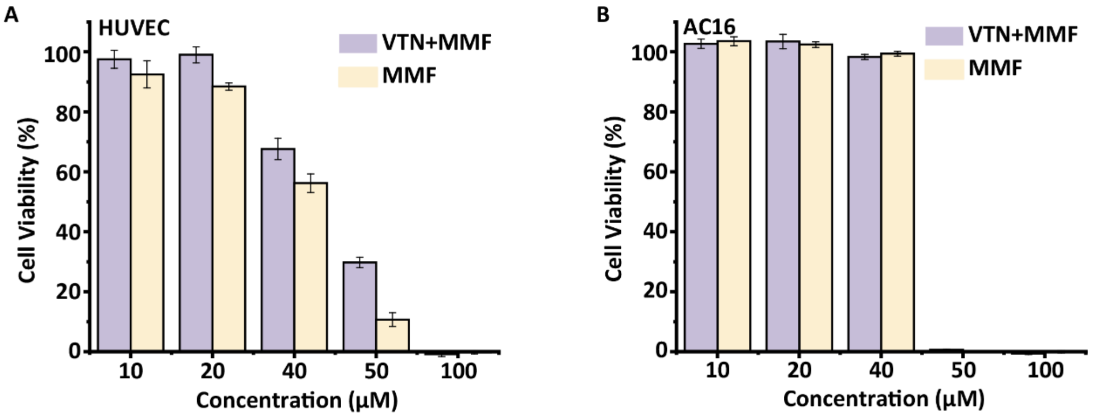

Figure S26. The effects of co-treatment with VTN and MMF on (A) HUVEC and (B) AC16 cell viability.

Table S1. Serum biochemical parameters of Balb/c mice.

	**PBS**	**VTN**	**VDN**
WBC	5.65±0.49	5.315±0.28	5.005±1.14
Neu	13.25±1.77	21.15±11.95	26.75±10.82
Lym	80.8±0.99	71.95±12.66	62.9±13.29
Mon	3.9±1.13	3.75±0.64	6.95±0.35
Eos	1.8±0.28	1.35±1.63	3.15±2.47
Bas	0.25±0.07	1.8±1.56	0.25±0.35
Neu#	0.745±0.04	1.14±0.69	1.4±0.85
Lym#	4.57±0.45	3.805±0.47	3.075±0.05
Mon#	0.225±0.09	0.205±0.05	0.345±0.06
Eos#	0.095±0.01	0.07±0.08	0.17±0.16
Bas#	0.015±0.01	0.095±0.08	0.015±0.02
RBC	5.24±0.30	4.675±0.08	4.48±0.04
HGB	128±9.90	110±2.83	110±1.41
HCT	21.65±1.63	21.4±0.42	20.15±1.34
MCV	41.3±0.71	45.7±0.14	45±3.54
MCH	24.45±0.49	23.55±0.21	24.55±0.07
MCHC	591±1.41	514.5±3.54	547.5±44.55
RDW-CV	15.45±2.05	23.2±0.42	20.6±5.37
RDW-SD	26.9±3.96	44.35±0.49	39.15±12.80
PLT	526.5±85.56	827.5±72.83	657.5±139.30
MPV	5.85±0.21	5.5±0.00	5.85±0.35
PDW	14.65±0.07	15±0.00	14.8±0.28
PCT	0.3085±0.06	0.4565±0.04	0.384±0.06

Table S2. Heart and Lung Transplantation (ISHLT) grading system.

**Score**	**Assessment**
**0**	No rejection.
**1**	Mild interstitial or perivascular infiltrate without necrosis.
**2**	Focal interstitial or perivascular infiltrate with necrosis.
**3**	Multifocal interstitial or perivascular infiltrate with necrosis
**4**	Widespread infiltrate with hemorrhage and/or vasculitis.
